# Pan-cancer analysis reveals technical artifacts in TCGA germline variant calls

**DOI:** 10.1186/s12864-017-3770-y

**Published:** 2017-06-12

**Authors:** Alexandra R. Buckley, Kristopher A. Standish, Kunal Bhutani, Trey Ideker, Roger S. Lasken, Hannah Carter, Olivier Harismendy, Nicholas J. Schork

**Affiliations:** 1 Biomedical Sciences Graduate Program, University of California, San Diego, La Jolla, CA USA; 2 J. Craig Venter Institute, La Jolla, CA USA; 3 Bioinformatics and Systems Biology Sciences Graduate Program, University of California, San Diego, La Jolla, CA USA; 4 Division of Medical Genetics, Department of Medicine, University of California San Diego, La Jolla, CA USA; 5 Moores Cancer Center, University of California San Diego, La Jolla, CA USA; 6 Cancer Cell Map Initiative (CCMI), University of California San Diego, La Jolla, CA USA; 7 Microbial Genomics Program, J. Craig Venter Institute, La Jolla, CA USA; 8 Division of Biomedical Informatics, Department of Medicine, University of California San Diego, La Jolla, CA USA; 9 The Translational Genomics Research Institute, Phoenix, AZ USA

**Keywords:** Cancer genomics, TCGA, Cancer germline, Whole exome sequencing, Variant calling, GATK, Batch effects, Whole genome amplification, Variant annotation, Genetic association testing

## Abstract

**Background:**

Cancer research to date has largely focused on somatically acquired genetic aberrations. In contrast, the degree to which germline, or inherited, variation contributes to tumorigenesis remains unclear, possibly due to a lack of accessible germline variant data. Here we called germline variants on 9618 cases from The Cancer Genome Atlas (TCGA) database representing 31 cancer types.

**Results:**

We identified batch effects affecting loss of function (LOF) variant calls that can be traced back to differences in the way the sequence data were generated both within and across cancer types. Overall, LOF indel calls were more sensitive to technical artifacts than LOF Single Nucleotide Variant (SNV) calls. In particular, whole genome amplification of DNA prior to sequencing led to an artificially increased burden of LOF indel calls, which confounded association analyses relating germline variants to tumor type despite stringent indel filtering strategies. The samples affected by these technical artifacts include all acute myeloid leukemia and practically all ovarian cancer samples.

**Conclusions:**

We demonstrate how technical artifacts induced by whole genome amplification of DNA can lead to false positive germline-tumor type associations and suggest TCGA whole genome amplified samples be used with caution. This study draws attention to the need to be sensitive to problems associated with a lack of uniformity in data generation in TCGA data.

**Electronic supplementary material:**

The online version of this article (doi:10.1186/s12864-017-3770-y) contains supplementary material, which is available to authorized users.

## Background

Cancer research to date has largely focused on genetic aberrations that occur specifically in tumor tissue. This is not without reason as tumor formation is driven to a great degree by somatically-acquired changes [1]. However, the degree to which germline, or inherited, DNA variants contribute to tumorigenesis is unknown. While it has been clearly demonstrated that germline variation increases cancer risk in overt and rare familial cancer predisposition syndromes, the contribution of germline variation to more common and sporadic cancer risk is unclear and highly debated [1, 2]. It is likely that inherited germline variation in fundamental molecular processes, such as DNA repair, can create a more permissive environment for tumorigenesis and shape tumor growth in some individuals [3–5]. It is also likely that variation in the host germline genome can act synergistically with acquired somatic mutations to shape the way in which tumors grow and ultimately manifest.

There is a growing interest in better understanding the contribution of germline variation to cancer risk and tumor phenotypes [6, 7]. The most extensive pan-cancer germline study to date identified associations between deleterious germline variation in known cancer predisposing genes and both age of onset and somatic mutation burden [6]. Lu et. al demonstrated that inherited variants can increase risk of developing cancer, as well as influence tumor growth and overall phenotypic features. Similar results were found in a study of bialleleic mismatch repair deficiency (bMMRD). It is known that bMMRD predisposes to childhood cancer, but it was further demonstrated that acquisition of somatic mutations in polymerase genes (*POLE*, *POLD1*) led to a hypermutated phenotype in childhood brain tumors [8]. This demonstrates a synergistic interaction between germline variation and somatic mutation. A comprehensive study of breast cancer whole genomes identified a somatic copy number profile signature associated with *BRCA1* inactivation [9]. Interestingly, this profile was associated with either inactivation of *BRCA1* in the tumor via mutation or promoter hypermethylation, or via inherited germline variants. This shows that somatic mutation and germline variation can both influence tumor phenotype.

We chose to use the whole exome sequence (WXS) data from TCGA to investigate the role of germline variation in shaping tumor phenotypes. TCGA is an attractive dataset for this purpose as there are paired tumor normal data for many cancer types. We took a pan-cancer approach for two reasons: 1. increased sample size and therefore increased power to detect associations of small effect size; and 2. cancers of disparate origin may share common features which would be overlooked in a cancer type-specific analysis [10]. For example, germline mutations in *BRCA1*/*2* are most commonly studied in breast and ovarian cancer, but have also been shown to increase risk for stomach and prostrate cancer [11]. Further, germline *BRCA2* mutations have been associated with a distinct somatic mutational phenotype and an overall increased somatic mutation burden in both prostrate and breast cancer [6, 9, 12]. To our knowledge, a comprehensive germline analysis of all cancer types available in TCGA has not been performed. Thus other cross-cancer germline associations likely remain to be discovered.

In an ideal dataset, a single protocol should be used for processing all samples. Unfortunately, this is unrealistic in large public datasets like TCGA in which samples are collected over time and across many data centers. Since its inception in 2005, TCGA has collected data on 11,000 patients from 20 collaborating institutions and generated sequence data from 3 sequencing centers [13]. Differences in sample collection and processing across centers could lead to batch effects, or variation in the data due to a technical factor that masks relevant biological variation [14]. Problems with batch effects can be amplified when analyzing samples across TCGA, since the number of methods used to collect samples increases with the number of cancer types. The Pan-Cancer Analysis Project has recognized this and aims to generate a high quality dataset of 12 TCGA cancer types, taking care to identify and minimize technical artifacts [10].

While extensive curated somatic data are available from TCGA, germline information is currently only available in raw form, under controlled access. Therefore, we first had to develop and execute a variant calling pipeline on the raw normal tissue sequence data. As a main goal of our variant calling analysis is to create a cohesive, pan-cancer dataset, we chose to use the Genome Analysis Toolkit (GATK) joint calling approach [15, 16]. Joint calling is a strategy for variant calling in which read data are shared across samples, in contrast to single sample calling where genotype decisions are made based on reads from a single sample only. There are three major advantages of this approach: the ability to distinguish sites that are homozygous reference vs. those that have insufficient data to make a call, increased sensitivity to detect variant sites that are poorly covered in any individual sample but well covered when the cohort is considered as a whole, and the ability to use GATK’s statistical modeling approach to variation filtration, known as ‘variant quality score recalibration’ (VQSR).

Here we describe our experience calling germline variants from a large cohort of TCGA normal tissue WXS samples spanning 31 cancer types. Specifically, we were interested in cataloguing sources of heterogeneity in sample preparation, identifying batch effects in our variant calls, and determining methods to reduce or control for technical noise. Our finding reveals a critical artifact introduced by preparation of DNA samples through whole genome amplification, leading to false positive LOF indels. The study therefore highlights the importance of quality control at all stages of the variant calling process and suggest that pan-cancer analysis with TCGA data be approached with caution.

## Results

### Technical heterogeneity in TCGA WXS Data Generation

**Fig. 1:**
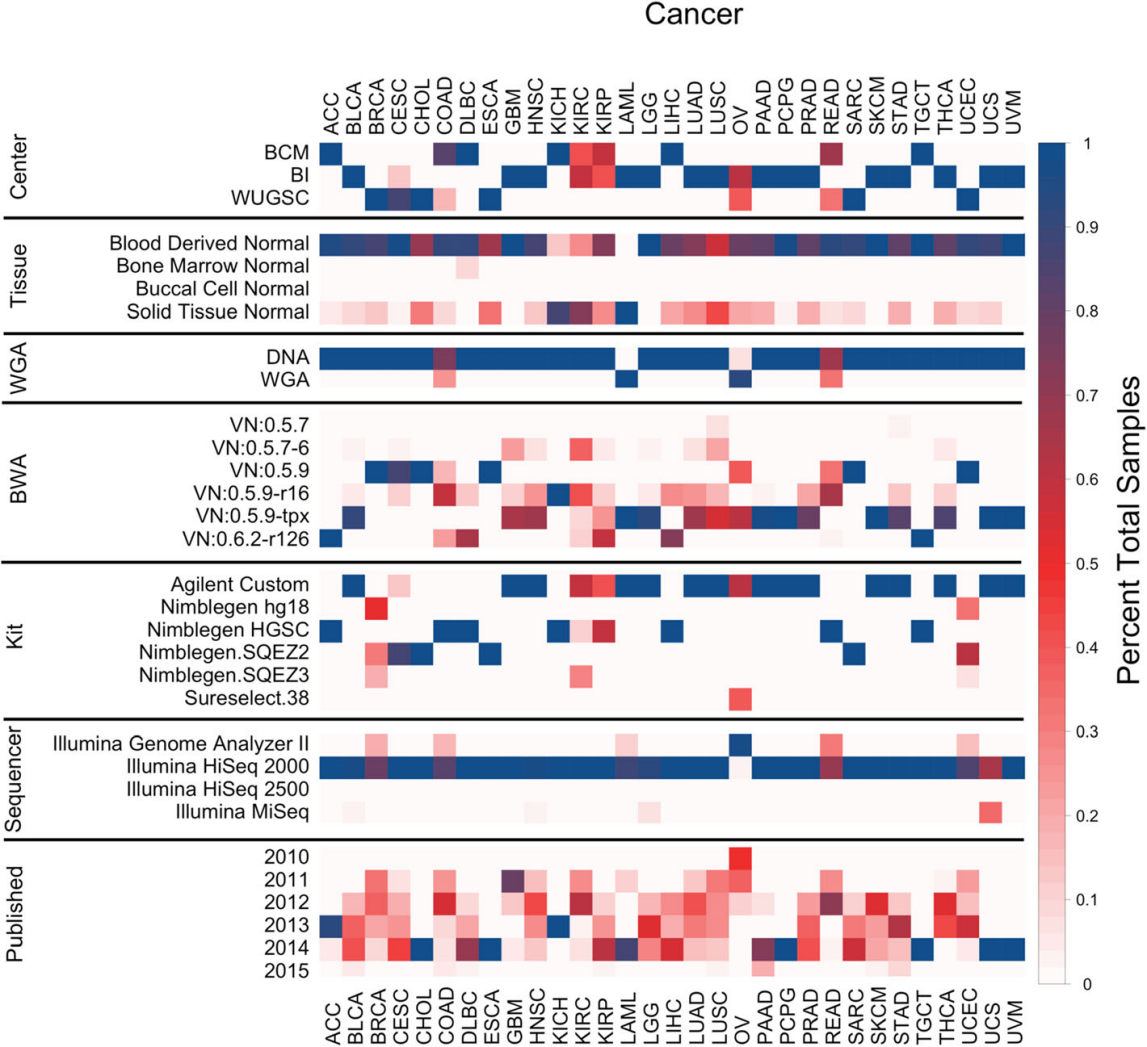
Overview of technical covariates for pan-cancer samples. For each covariate and cancer type, color represents the fraction of total samples. Fraction of total samples sums to 1 for each covariate and cancer type. Red indicates higher heterogeneity. Year first published included for context. TCGA cancer abbreviations: ACC, adrenocortical carcinoma; BLCA, bladder urothelial carcinoma; BRCA, breast invasive carcinoma; CESC, cervical squamous cell carcinoma and endocervical adenocarcinoma; CHOL, cholangiocarcinoma; COAD, colon adenocarcinoma; ESCA, esophageal carcinoma; GBM, glioblastoma multiforme; HNSC, head and neck squamous cell carcinoma; KICH, kidney chromophobe; KIRC, kidney renal clear cell carcinoma; KIRP, kidney renal papillary cell carcinoma; LAML, acute myeloid leukemia; LGG, brain lower grade glioma; LIHC, liver hepatocellular carcinoma; LUAD, lung adenocarcinoma; LUSC, lung squamous cell carcinoma; OV, ovarian serous cystadenocarcinoma; PAAD, pancreatic adenocarcinoma; PCPG, pheochromocytoma and paraganglioma; PRAD, prostate adenocarcinoma; READ, rectum adenocarcinoma; SARC, sarcoma; SKCM, skin cutaneous melanoma; STAD, stomach adenocarcinoma; TGCT, testicular germ cell tumors; THCA, thyroid carcinoma; UCEC, uterine corpus endometrioid carcinoma; UCS, uterine carcinosarcoma; UVM, uveal melanoma

We obtained TCGA WXS data from CGhub in the form of reads aligned to the human reference genome (BAM files) [17]. From the BAM files and available metadata we identified seven technical sources of variation in the way the sequence data were generated: tissue source of normal DNA, exome capture kit, whole genome amplification of DNA prior to sequencing (WGA), sequencing center, sequencing technology, BWA version, and capture efficiency (C20X) (Additional file 1: Figure S1, Additional file 2). We found substantial variation existed within and between cancer types with respect to these technical factors (Fig. 1). Some of these technical factors were found to be highly associated with cancer type, such as use of Illumina Genome Analyzer II and ovarian cancer (OV), while others exhibited no clear relationship with cancer type, such as use of solid normal tissue as opposed to blood as a source of normal DNA. Relationships existed between pairs of technical factors as well, such as the Broad Institute’s exclusive use of a custom Agilent exome capture kit. All possible combinations of the first six technical factors produce 1152 unique workflows, of which only 44 were used to generate the TCGA data. This further demonstrates that relationships exist between technical factors. Of the 31 cancer types examined, only uveal melanoma (UVM) and testicular germ cell tumors (TCGT) had a uniform workflow for all samples (Additional file 1: Figure S1). These observations highlight the substantial heterogeneity in data generation across TCGA and importantly even within cancer types.

The technical factors can ultimately be divided into two categories: those that can be modified during processing of the sequence data (BWA version, target regions of a capture kit), and those that cannot be modified computationally (source of normal DNA, WGA, center, technology, capture efficiency). Six exome capture kits ranging in size from 33 to 64 MB were used to capture normal DNA for sequencing (Additional file 1: Table S2). As the goal of our variant calling pipeline was obtain a uniform set of variants across samples, we chose to restrict analysis to the intersection of the capture regions. The area hereby excluded consists largely of exon flanking regions. The intersection covers 97.7% of Gencode exons, thus for the purposes of studying protein-coding variation using the intersection of the kits leads to minimal loss of data (Additional file 1: Table S2) [18]. It has been shown that differences in capture efficiency and sample preparation protocols between exome kits can affect variant calls, even in regions common between kits [19]. Therefore, despite using the common capture region, the use of multiple capture kits may still introduce artifacts.

To assess the effect of heterogeneous BWA alignments on variant calls, we called variants on 345 of the TCGA normal samples either using the provided BAM (OldAlign) or stripping and realigning reads to GRCh37 using BWA MEM v.0.7.12 (NewAlign). The overall raw discordance rates between the two sets of variants was 5%, which is in the expected range for different alignment protocols (Additional file 1: Figure S3) [20]. Indel calls were noticeably more discordant, consistent with the specific challenges and notorious variability of indel calling [21]. Interestingly, the discordance rate was correlated with BWA version used to generate the BAM file in CGhub, with older versions displaying more discordance. This effect can largely be reduced by applying VQSR filters, which decreases overall discordance from 5 to 3% (Additional file 1: Figure S4). Greater discordance between variant calling pipelines has been observed in repetitive regions of the genome, and in accordance with this we reduce overall discordance to 1.7% with the removal of repetitive regions from analysis (Additional file 1: Figure S3) [22]. As no set of true positive variants exists for TCGA samples, we cannot determine whether realigning BAM files produces more accurate calls. Given the computational cost of realignment, and that discordance can be mitigated by filtering variants and masking repetitive regions of the genome, we proceeded with variant calling using the provided BAM files.

Functional annotation of the 1,093,501 variants in the final VCF predicted 625,365 missense; 371,754 silent; 24,455 nonsense; 2968 splice site; 553 stoploss; 46,280 frameshift indels and 22,126 in-frame indels in 9618 samples. For initial quality control we performed principal component analysis (PCA) to identify the most significant sources of variation in the variant calls. PCA on common variants showed that the first two principal components stratified samples by self-reported race and ethnicity, indicating that the largest source of variation is ethnic background and not technical factors (Additional file 1: Figure S5). To assess the quality of the calls, we measured the fraction of variants also present in the ExAC database [23]. We expect a high degree of overlap between our calls and ExAC, as the ExAC v0.3.1 dataset includes germline variants from 7601 TCGA individuals. Overall 88.56% of the variant calls were present in ExAC, with SNVs showing higher overlap than indels (89.91% vs. 53.94%). Based on these results, we concluded the variant calls were free of overt technical artifacts and proceeded to the next stage of analysis.

### Impact of technical heterogeneity on loss of function variants

**Fig. 2:**
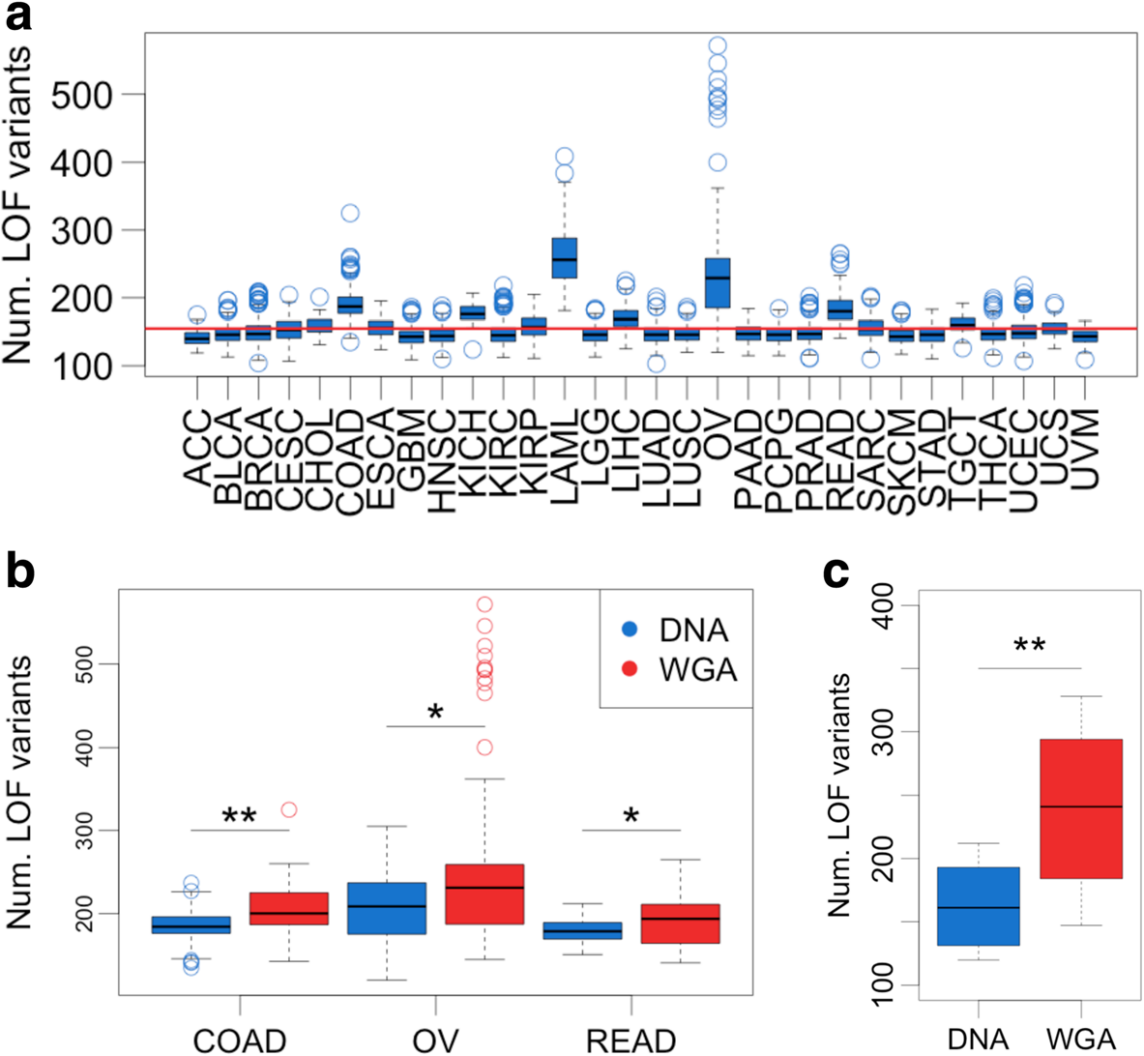
WGA increases LOF variant burden. **a** LOF variant burden includes both SNV and indels. Red line indicates expected LOF burden from ExAC (155). **b** Individual LOF variant burden in cancers with WGA samples plotted by WGA status..* = Wilcoxon rank sum test *p* < 0.05, ** = Wilcoxon rank sum test *p* < 0.001. **c** Individual LOF variant burden in *n* = 13 samples that have both DNA and WGA samples available. ** = Wilcoxon paired rank sum test *p* < 0.001

There is great interest in understanding how inherited impaired functionality of cancer-relevant pathways shapes tumor phenotypes, as has been previously demonstrated for bMMRD and *BRCA1* germline mutations [6, 8, 9]. To identify germline variation likely to disrupt function of genes, we used VEP and LOFTEE to predict LOF variants in this cohort [24]. We observed a median 150 LOF per sample across our entire cohort, consistent with the ExAC findings (Fig. 2a) [23]. However, two cancer types, acute myeloid leukemia (LAML) and OV deviate significantly from this expected value, with individuals with these cancers having up to 500 LOF germline variants. This suggests an artifact was manifesting in rare LOF variants that was not identified by PCA on common variants. Notably this effect is specific to LOF indels, in contrast to LOF SNVs that are distributed more uniformly across cancer types (Additional file 1: Figure S6).

We used Analysis of Variance (ANOVA) to assess the contribution of each technical factor to individual LOF variant burden. Initial analysis showed that source of normal control DNA and sequencing technology were not significantly associated with LOF variant burden, and that capture kit was highly collinear with sequencing center. Therefore, we limited subsequent analysis to sequencing center, BWA version, WGA, and C20X. It is known that LOF variant burden varies between ethnic groups, thus we include self-reported race as a covariate in this analysis as a reference point for expected variation [23]. All technical factors combined explain less than 1% of the variance in LOF SNV burden, indicating SNVs are largely unaffected by technical variation. In contrast, 59% of variation in LOF indel burden was explained by technical factors, with WGA alone explaining over 50% (Table 1).

**Table 1:** Variance in LOF SNV and indel burden explained by technical covariates

	Sum. Sq.	Df	F value	*P* value	% Var. Exp.
LOF SNV					
C20X	1785.49	1	52.95	3.72e^-13^	0.0056
WGA	156.89	1	4.65	3.10e^-02^	0.0005
Center	716.08	2	10.61	2.48e^-05^	0.0023
BWA	79.59	5	0.47	7.97e^-01^	0.0003
RACE	30698.90	5	182.10	1.33e^-184^	0.0973
Residuals	281966.85	8363			0.8940
LOF Indel					
C20X	52930.43	1	153.90	4.95e^-35^	0.0072
WGA	3744887.28	1	10888.62	0.0000	0.5080
Center	383585.43	2	557.65	4.53e^-228^	0.0520
BWA	169507.9	5	98.57	2.76e^-101^	0.0229
RACE	146904.86	5	85.42	7.59e^-88^	0.0199
Residuals	2876257.21	8363			0.3900

ANOVA results table

*Sum. Sq*. Sum of Squares; *Df* Degrees of Freedom; % *Var. Exp*. Percent variance explained by each factor (factor Sum. Sq./total Sum. Sq.)

WGA samples have a higher LOF variant burden with a median 201 LOF variants per WGA sample. Four cancer types contain samples that underwent WGA: colon adenocarcinoma (COAD) (26% WGA), rectum adenocarcinoma (READ) (33% WGA), OV, (92% WGA) and LAML (100% WGA) (Fig. 1). Analyzing cancer types containing both amplified and non-amplified DNA samples, we observed that WGA samples had a significantly higher LOF variant burden (Fig. 2b), further suggesting that WGA rather than cancer type is the main source of bias. The cohort contains 13 individuals with both amplified and non-amplified DNA samples. We observed a 1.5 fold increase in LOF variant burden in amplified samples relative to non-amplified samples from the same individuals (*p* = 0.0002 by paired Wilcoxon Signed Rank test) (Fig. 2c), suggesting that WGA prior to sequencing leads to an artificially inflated number of predicted LOF variants.

To determine whether our choice not to realign BAM files contributed to the observed WGA effect, we calculated LOF variant burden in our NewAlign and OldAlign cohort using the same protocol. Realignment of the sequence data with BWA MEM increased the number of LOF calls per individual but overall LOF burden was highly correlated (Pearson *R* ^2^ = 0.95) (Additional file 1: Figure S7). WGA explained a significant amount of variance in LOF variant burden in both NewAlign and OldAlign samples (Additional file 1: Figure S7). Thus we can conclude that realignment does not remove WGA artifacts observed in our variant calling pipeline.

### Characterizing WGA artifacts

**Fig. 3:**
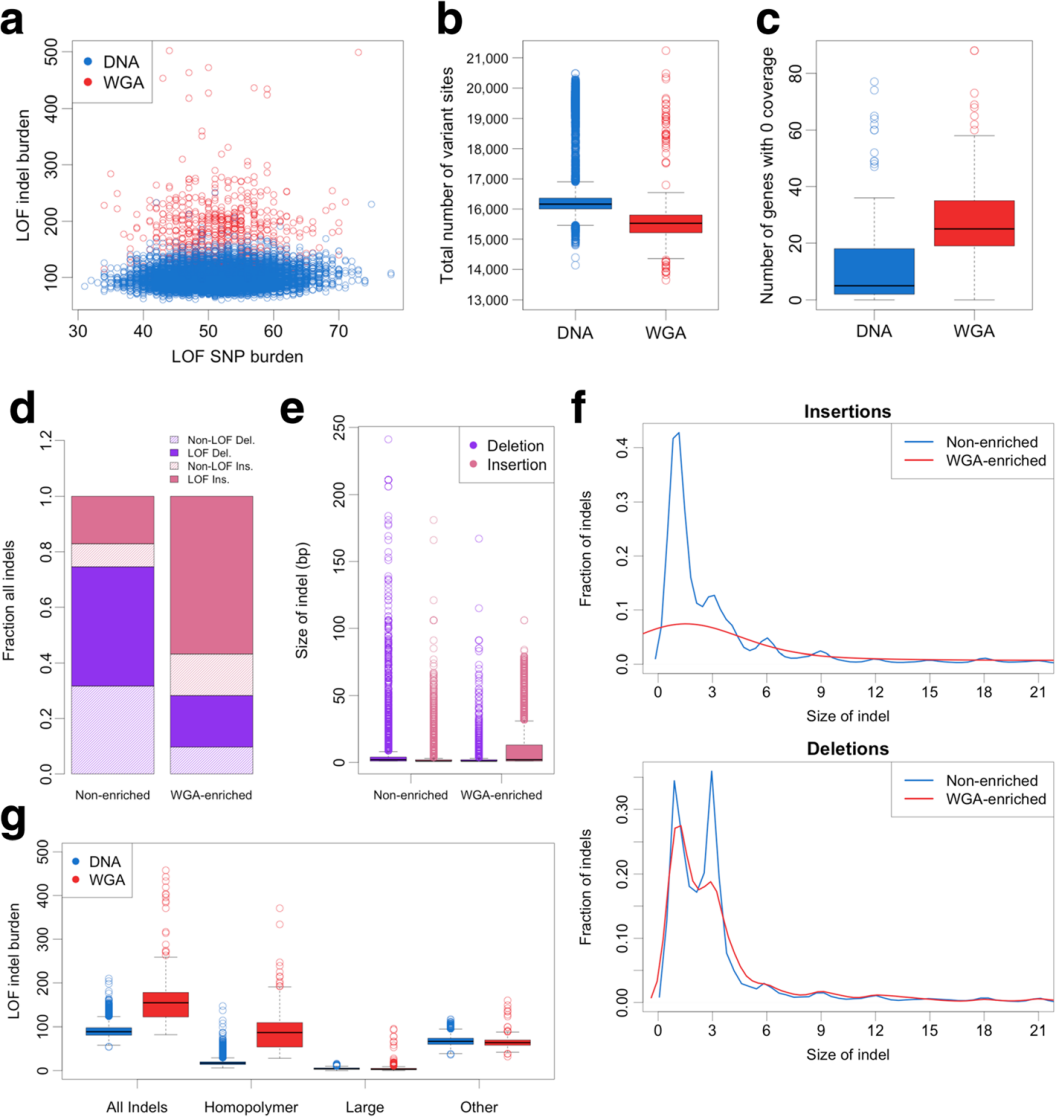
Characteristics of variant calls in WGA samples. **a** Individual LOF indel burden vs. individual LOF SNV burden. Color indicates WGA status. **b** Total number of variant calls plotted by WGA status. **c** Number of genes with 0 read depth across 16,824 genes. **d** Fraction of insertions and deletions in *n* = 5654 WGA-enriched and *n* = 34,880 non-enriched indels. Shading indicates LOF status. **e** Size in base pairs of WGA-enriched and non-enriched indels. **f** Density plot showing distribution of insertion and deletion size for WGA-enriched and non-enriched indels. **g** Individual burden of LOF indels for all indels, homopolymer + indels, indels 15 base pairs or longer, and other indels. Color indicates WGA status. Indel burden calculated using GATK VQSR TS99 filter

Having demonstrated that WGA is associated with increased LOF variant burden, we sought to characterize WGA samples more deeply. We observe that WGA samples have an excess of LOF indels while LOF SNV burden appears unaffected, as expected from the ANOVA results (Fig. 3a). Interestingly, WGA samples had fewer variants overall, due more variable coverage depth over the capture regions (Fig. 3b, Additional file 1: Figure S8). Read depth was highly variable across genes in WGA samples with an average depth of 165 X and standard deviation of 140 X (Additional file 1: Figure S8). As a consequence of this variable coverage, an average of 27 genes per sample had 0 coverage in WGA samples (Fig. 3c).

As indel variant calls are the source of inflated LOF variant burden in WGA samples, we next determined which indels are enriched in WGA samples using a one-way Fisher’s exact test. While it is impossible to distinguish errors from true indels definitively at this scale, indels that are a found at a significantly higher frequency in WGA samples relative to DNA samples are good candidates to be errors. The majority of WGA-enriched indels are insertions, and the ratio of insertions to deletions is skewed relative to non-enriched indel sites (Fig. 3d). Further, 75% of WGA-enriched indels are LOF relative to 60% of non-enriched indels (Fig. 3d). Upon examining the size of the indels in base pairs, we noticed that WGA-enriched insertions were larger than non-enriched insertions and their size distribution deviated from what is expected for coding indels (Fig. 3e,f). The length of indels in coding regions is frequently a multiple of three base pairs, due to natural selection acting to maintain the reading frame [25]. WGA-enriched insertions did not show this expected distribution, and thus are more likely to be LOF frameshift indels. As previously reported, LOF variants are enriched for sequencing errors, supporting our hypothesis that the excess LOF indels in WGA samples are technical artifacts [26].

We observe that the local sequence context surrounding WGA-enriched insertions has a higher GC content, and that G and C insertions are twice as frequent in WGA-enriched insertions than non-enriched insertions (Additional file 1: Figure S9, Table S7). This observation prompted us to look for homopolymer repeats in the sequence surrounding WGA-enriched indels. WGA-enriched indels occur in homopolymer repeats more frequently than non-enriched indels (Table 2). Further, indels that occur in homopolymer regions had an increased allele frequency in WGA samples relative to indels not in homopolymer regions, indicating that homopolymer indels are also more recurrent in WGA samples (Additional file 1: Table S8). We observe that WGA-enriched indels are larger on average and are frequently in homopolymer regions, but that these two characteristics are mutually exclusive. To better resolve the contribution of each of these indel types to WGA technical artifacts, we define three distinct categories of indels: homopolymer +, large, and all other indels (Table 2). Calculating individual LOF indel burden for each of these categories shows that the increased LOF indel burden observed in WGA samples is due to an excess of LOF homopolymer + indels (Fig. 3g).

**Table 2:** Fraction of WGA-enriched and non-enriched indels in three indel categories

	% Other Indels	% Homopolymer Indels	% Large Indels
WGA-enriched	47.78	27.13	25.07
Non-enriched	83.52	9.63	6.83

Homopolymer indels: indels with a 4 or more single base repeat directly proximal to the indel; Large indels: indels with 15 or more inserted or deleted bases. Other indels: all indels that don’t fit one of the previous criteria

The pan-cancer cohort contains 492 individuals with multiple germline WXS samples. Presumably, variants that are not concordant between repeated samples on the same individual are errors, and thus we used genotype discordance as a surrogate measure for variant calling error. In addition to the 13 individuals with paired normal WXS samples with and without amplification (denoted WGA:DNA), 44 individuals have paired normal WXS samples where both samples have been amplified (denoted as WGA:WGA) and 435 are paired samples without amplification (denoted DNA:DNA). We calculated genotype discordance between all repeated samples for SNVs and indels separately and observed a stepwise increase in discordance with amplification of one or both samples. This effect was most apparent in indels, with a median 59.9% indel discordance between repeated WGA:WGA samples (Additional file 1: Figure S10). Calculating indel discordance using the indel categories previous defined reveals that discordance between WGA samples is highest for homopolymer + indels, lower for large indels, and similar to DNA samples for other indels (Additional file 1: Figure S10). This demonstrates that WGA errors manifest as small indels in homopolymer regions and large indels with no clear sequence context bias.

WGA by multiple displacement amplification (MDA) is known to create chimeric DNA rearrangements, which manifest in the sequence data as reads with sequence from noncontiguous portions of the reference genome (Additional file 1: Figure S11) [27]. To determine if chimeric reads were responsible for the large indels in WGA samples, we used BLAST to align the inserted and deleted sequences of large indels to the reference genome [28]. We observe that 86% of WGA-enriched large insertion sequences have a BLAST match, whereas only 10% WGA-enriched large deletions and non-enriched large indels have a BLAST match (Additional file 1: Table S9). Further, the BLAST matches for WGA-enriched insertions were predominantly within 2 kb of the indel start position which is in accordance with the mechanism of MDA chimeric rearrangements (Additional file 1: Figure S12). Thus, the large indels we observe in WGA samples can be explained by known MDA artifacts (Additional file 1: Figure S11). Small indels in homopolymer regions may occur by the same mechanism, as it has been shown that the majority of MDA chimeric junctions occur in regions of short complimentary sequence [27]. The small homopolymer indel errors may also be due to known difficulties of calling indels in homopolymer regions, which is exacerbated with amplification [29].

### Filtering artifactual LOF variant calls

**Fig. 4:**
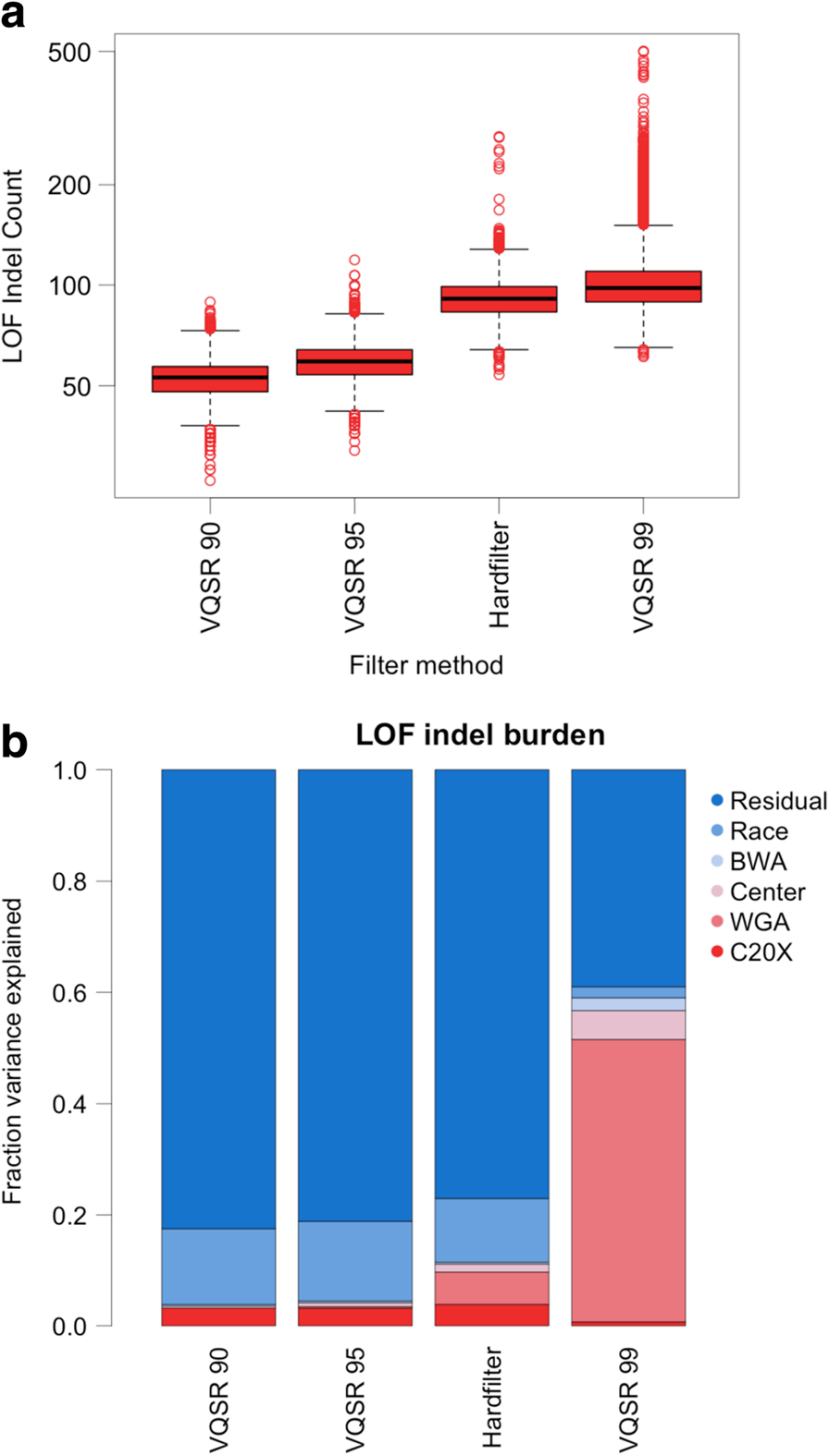
A comparison of indel filtering strategies. **a** Individual LOF indel burden for all indel filter methods in order of decreasing stringency. **b** Percent of variation in individual LOF indel burden explained by technical covariates for each filter method

We next sought an appropriate filter to remove artifactual LOF variant calls in WGA samples. As SNV calls were largely robust to technical artifacts, we focused on filtering indels specifically (Additional file 1: Figure S6). We used two strategies available from GATK: 1) Statistical model filtering using VQSR with increasing stringency cutoffs (99, 95, 90%), and 2) Heuristic filtering (Hardfilter) based on fixed thresholds (QD > 2, FS < 200, ReadPosRankSum > -20), for a total of four filtering approaches [16]. The four filters varied in stringency, resulting in a median individual LOF indel burden ranging from 53 to 98 across methods (Fig. 4a and Additional file 1: Figure S13). To assess the efficiency of each filter to remove technical artifacts, we performed an ANOVA analysis as described in Fig. 2 for each filtering approach, including the initial filter (GATK VQSR 99) as a reference (Fig. 4b). VQSR 90 and VQSR 95 reduced technical artifacts to a similar degree, whereas VQSR 99 and Hardfilters performed poorly (Additional file 1: Figure S14A, Table S10).

Variant filtering is a balance between removing likely false positive signal while retaining true positive signal. Using VQSR 99 we observe an individual LOF variant burden similar to that reported in the ExAC database, while all other methods produce lower LOF burden than expected (Additional file 1: Figure S14A) [23]. Therefore, while more stringent filtering approaches can reduce technical artifacts, they do so at the cost of losing likely true positive indels. Without a way to manually validate a large number of rare indel variant calls, it is impossible to exactly measure false positives rates for our filter approaches.

Instead, we once again used the repeated samples in our cohort to identify likely true positives (indels concordant between repeated samples) and likely false positives (indels discordant between repeated samples). We assessed filter quality using three measures: the fraction of discordant indels removed by the filter, the fraction of concordant indels removed by the filter, and the fraction of indels overlapping the ExAC database. The stringency of each filter was measured as the total number of LOF indel sites and the median individual indel LOF burden when each filter was applied (Table 3).

**Table 3:** Metrics of filter stringency and efficacy

Filter	LOF indel sites	Median LOF indel burden	Fraction discordant indels removed	Fraction concordant indels removed	Indel overlap with ExAC
VQSR 90	6212	53	0.8667	0.4514	0.7079
VQSR 95	9177	59	0.8064	0.3760	0.6776
Hardfilter	24212	91	0.3600	0.0210	0.3527
VQSR 99	26134	98	0.2763	0.1100	0.5394

GATK VQSR 90 is the only filter capable of eliminating the significant association between WGA and LOF indel burden, however; it does so at the cost of over 75% of all LOF indel sites (Additional file 1: Table S10). From this we can conclude that WGA artifactual indels closely resemble true indels, preventing VQSR from selectively removing artifactual indels

### Consequences of technical artifacts on genetic associations

To determine how sensitive association results are to filtering method, we tested for association between germline LOF variant burden and cancer type using different filtering approaches. We took an ‘one vs. rest’ approach with our samples using all cancers except the cancer of interest as a control. Thus, we tested for enrichment of LOF germline variants in one cancer type as compared to other cancers, which is different than other studies that have used control cohorts [6]. Our rationale for using this approach was to minimize heterogeneity that would be introduced by including control samples collected in different studies. We chose to highlight the results only from OV for two reasons. First, it is established that *BRCA1*/*2* germline variants are enriched in OV so the OV—*BRCA1*/*2* association can be used as a positive control, and second virtually all OV samples have been amplified and are confounded with WGA artifacts [6, 30, 31].

**Fig. 5:**
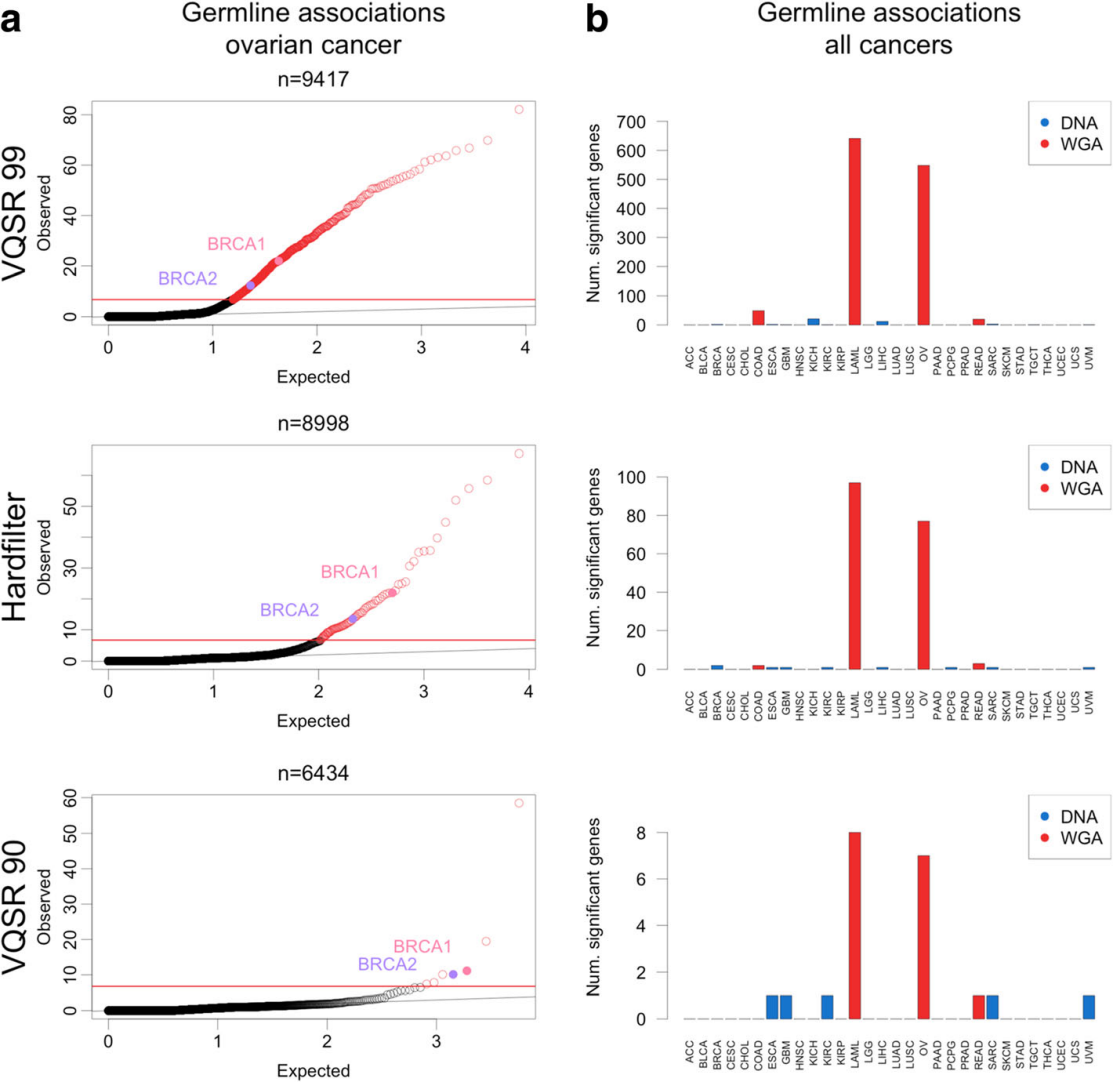
Association testing between germline LOF variant burden and cancer type. **a** Quantile-quantile plots from logistic regression association testing between germline LOF burden and ovarian cancer for three indel filter methods. n = number of genes tested. Red line indicates significant cutoff and red points indicate associations significant *p* < 1.61 × 10^-7^. *BRCA1*/*2* associations highlighted. **b** Number of significant cancer type - gene associations in each cancer type for three indel filter methods. Color indicates cancer types with WGA samples

Quantile-quantile plots from logistic association tests for three indel filter methods are shown in Fig. 5a. It was immediately apparent that our initial filtering approach (VQSR 99) produced an excess of significant associations even above a strict Bonferroni multiple hypothesis correction (Fig. 5b). True associations are mixed with false associations due to WGA artifacts in LOF indel calls. Increasing the stringency of indel filtering reduced noise due to technical artifacts while retaining a putative true positive *BRCA1*/*2* association signal. Stringent filtering removes noise at the cost of reducing potential signal, as evidenced by the decreased number of genes that can be tested for association. This inflation in significant associations was only observed in cancers containing WGA samples, and persisted, albeit to a far lesser extent, even with the most stringent filter (Fig. 5b). Supporting the idea that some of the associations in WGA cancer types are false, only two of the significant genes (*BRCA1*/*2*) in OV and none in LAML are genes where germline variation is known to be associated with cancer risk [32].

We observe that an unusually high fraction of significantly associated genes were shared between LAML and OV, with 69, 55, and 25% of significant genes shared for VQSR filters TS99, TS95, and TS90, respectively (Additional file 4: Table S11). Having demonstrated that LOF indels occur at a high allele frequency in homopolymer regions in WGA samples, we calculated the number of homopolymer regions in these shared genes. We observe that shared genes have a higher G/C homopolymer content compared to all genes tested (Additional file 1: Figure S15). Further we see a stronger correlation between LOF indel burden and homopolymer content in WGA samples than in DNA samples (Additional file 1: Table S12). Taken together, we can conclude that the high fraction of shared genes between LAML and OV is driven by high allele frequency LOF indels in homopolymer regions. LOF indel calls are more prone to batch effects than LOF SNVs, therefore we repeated the association test limiting to LOF SNVs only. While this reduces the excess number of significant associations, the analysis was underpowered to detect the true positive *BRCA1*/*2*—OV association (Additional file 1: Figure S16). These results demonstrate that technical artifacts can lead to spurious associations and highlight the difficulty of correcting for artifacts in a pan-cancer analysis when technical factors are highly correlated with the phenotype being tested (Fig. 1).

## Discussion

We identified sources of technical variation in LOF variant calls from TCGA germline WXS data. Overall SNV calls were more robust to technical factors than indel calls. We found the strongest association between amplification of DNA prior to sequencing and an excess of LOF indel calls. Other factors tested were found to be significantly associated with both LOF SNV and LOF Indel burden, but explain little of the total variance in LOF variant burden when appropriate filters are applied (Table 1 and Fig. 4b). The factor explaining the most technical variation in total LOF variant calls after filtering is capture efficiency (C20X). It is likely that poor coverage over common capture regions, perhaps due to the different capture technologies used, decreased the ability to assign genotypes in some samples. Joint calling distinguishes sites with insufficient coverage to make a genotype call from those with adequate coverage for calling a homozygous reference genotype. Therefore, while C20X is a significant factor in the simple burden analyses performed here, a more sophisticated burden testing approach that can accommodate missing genotype values should mitigate this technical artifact.

Difficulty producing reliable variant calls in WGA exome samples has been previously reported [19, 33]. Inaccurate read alignment has been identified as a main contributor to spurious calls in WGA samples. However, even with an alignment protocol optimized for WGA samples it is still estimated that 7% of variant calls in WGA samples are artifactual [19]. Previous work comparing amplified and non-amplified DNA obtained from the same biological sample report higher variant call discordance in indels compared to SNVs, similar to what we observe [33]. These studies conclude that overall concordance between amplified and non-amplified samples is satisfactory; however, neither examined the impact of WGA on deleterious variants. Here we have demonstrated that errors introduced by WGA manifest as rare frameshift indels that are difficult to distinguish from true rare deleterious variation. We further demonstrated that the WGA indel errors we observe are in accordance with known errors and biases that occur due to MDA, and provide a mechanism by which MDA chimeric reads lead to erroneous indel calls (Additional file 1: Figure S11). In addition to drawing attention to batch effects in TCGA sequence data, our study also provides valuable insight into potential pitfalls of calling indels in sequence data generated from MDA.

Simultaneous to our investigation, the genomic data commons (GDC) has called somatic mutations on TCGA tumor sequence data using four different pipelines and discovered an excess of insertion mutations in tumor samples with amplified DNA [34, 35]. This validates our findings in the orthogonal process of somatic mutation calling. Further, GDC only reports this observation for the MuTect2 pipeline, which combines aspects of the original MuTect algorithm and GATK’s ‘HaplotypeCaller’ [36]. As WGA artifacts have thus far only been observed in GATK-derived variant callers, it is possible that these artifacts are specific to the GATK pipeline. An alternate method of variant calling could reduce or eliminate WGA errors, but this issue is still problematic as GATK is one of the most commonly used variant callers for large datasets such as ExAC and gnomAD [23].

While joint calling is the approach recommended by GATK, with the exception of one paper from our lab exploring the impact of genetic background on joint calling, to our knowledge there has not been a published systematic comparison of joint calling vs. single sample calling with GATK on a gold standard dataset to quantify the advantages of joint calling [37]. GATK’s joint calling approach is not without problems. Greater accuracy for the group as a whole comes at the cost of loss of singleton variants from any given sample. Another complicating factor unique to joint called samples are multi-allelic sites, or sites where multiple alternate alleles are found in the population genotyped. Relatively few sites in our VCF were multi-allelic (3%, or 30,620 sites), but these sites contain 4947 high-confidence LOF variants (11% of all LOF variants), indicating the importance of correct multi-allelic site parsing. Multi-allelic sites additionally pose a problem when filtering reliable from unreliable variants. With current tools for filtering VCFs, it is only possible to filter at the site level, meaning at multi-allelic sites all alleles will either be included or excluded by the filter. Further, in the version of GATK used for this analysis (v3.5), quality annotations for a site are calculated using all alternate reads without distinguishing between alleles. Therefore it is possible for low quality alternate alleles to pass filter at multi-allelic sites if high quality alternate alleles are present at the same site.

## Conclusions

Our work shows that amplification of DNA prior to sequencing resulted in an excess of predicted damaging indel variants. In our dataset, we find that using VQSR TS90 can eliminate the significant association between WGA and LOF indel burden, but it appears false associations persist in our association analyses (Fig. 5b, Additional file 1: Table S10). Thus, we find removal of WGA samples to be the only option to fully eliminate batch effects in our dataset. It is possible WGA indel artifacts could be eliminated in WGA samples using a different variant calling approach perhaps sensitive to MDA induced errors. The GDC has worked to optimize MuTect2 parameters for WGA samples, and their methods could potentially be applied to germline variant calling [34]. We suggest that variant calling in these samples should be handled with extra care.

TCGA is often thought of as a single dataset, but due to differences in sample collection and processing across the participating sites, should be thought of as a collection of studies. While we focused on the germline WXS sequence data, it is likely that batch effects are present in other data types. This has been recognized by the Pan-Cancer TCGA effort, although it is less often acknowledged in papers published on one or few cancer types [10]. There is heterogeneity even within cancer types in terms of sample preparation, such as in COAD and READ where roughly a third of germline WXS samples were prepared using WGA. Batch effects present in TCGA data can potentially confound even single cancer type analyses if not properly addressed. In terms of pan-cancer analysis, the correlation between certain technical factors and cancer types confounds analyses that use cancer type as the phenotype of interest, as we demonstrated in Fig. 5. We note that since the initiation of our analysis, the raw TCGA sequence data have moved to the GDC [35]. The GDC has realigned the sequence to the current reference genome (GRCh38 .d1.vd1) using a standardized pipeline to harmonize the BAM file. Although this will eliminate one source of variation (BWA version), it only serves to remind researchers how sensitive data analyses might be to non-standardized data collection protocols, especially in the context of the TCGA data, as our study makes clear. Analyses of large, extant data sets will continue to grow and impact biomedical research, with many in the community committed to pointing out the need for care in interpreting the results and impact of those analyses [14, 38, 39].

## Methods

### Cohort

Approval for access to TCGA case sequence and clinical data were obtained from the database of Genotypes and Phenotypes (dbGaP). We selected a total of 9618 normal tissue DNA samples with whole exome sequence data (Additional file 1: Table S1). We limited analysis to samples sequenced with Illumina technology and aligned to the GRCh37/hg19 reference genome.

### Germline Variant Calling

Aligned sequence data for normal samples in BAM file format and the accompanying metadata was downloaded from CGhub [17]. Individual samples were matched with the target regions for the exome capture kit used to generate the sequence data, and variant calling was limited to these target regions +/- 100 bp. SNVs and small indels were identified using the GATK v.3.5/v.3.4 best practices pipeline and a joint calling approach [15, 16]. The GATK pipeline includes two preprocessing steps to improve the quality of the BAM file. Local realignment of reads is performed in regions containing indels, and base quality scores are recalibrated to minimize known sources of score bias. ‘HaplotypeCaller’ was run on individual samples in gVCF output mode, producing an intermediate single sample gVCF to be used for joint genotyping. Running this pipeline on a single BAM from CGhub took approximately 15 compute hours and produced a 100 MB gVCF. Individual gVCFs were combined in groups of 100 and the final joint genotyping step was performed by chromosome on all 9618 samples as a single cohort. Following this joint genotyping step, all future analysis was limited to the intersection of all exome kit capture regions. The intersection of the kits covered 27 MB and 97.7% of Gencode v19 exons (Additional file 1: Table S2) [18]. GATK VQSR was run separately for SNVs and indels. VQSR learns from variant quality annotations using variants overlapping with vetted resources such as dbSNP and 1000 genomes as a truth set. VQSR filters are defined by the percentage of truth variants that pass filter, termed truth sensitivity (TS). For the initial analysis, SNVs were filtered at VQSR TS 99.5% and indels at VQSR TS 99.0%, as suggested by GATK documentation.

### PCA and Self-Report Ancestry Validation

PCA was performed jointly on the filtered pan-cancer VCF and HapMap genotype data from 1184 individuals using PLINK v1.90b3.29 [40, 41]. Multiallelic sites, rare variants (<1% AF), and sites with missing values were excluded from the pan-cancer VCF. A final variant set of 4376 SNPs was obtained by taking the union of the pan-cancer and HapMap variant calls, requiring 100% genotyping rate across all samples. To assess accuracy of self-report ancestry from TCGA clinical data, principle component (PC) loadings of TCGA samples and HapMap samples were compared. HapMap samples were clustered on PC 1 and PC 2 using the R package ‘flexclust’ and K-means clustering with k = 4 to roughly approximate the four major TCGA self-reported ancestry categories (White, Asian, Black, and Hispanic) (Additional file 1: Table S4) [42]. TCGA samples were assigned to one of these four clusters using the predict function and PC 1 and PC 2 loadings (Additional file 1: Table S5). Comparing self-reported ancestry to HapMap cluster membership showed 4% of TCGA samples had inaccurate self-reported ancestry (Additional file 3: Table S6).

### Annotation and BAM metrics

Putative LOF variants, defined here as stop-gained, nonsense, frameshift, and splice site disrupting, were identified using the LOFTEE plugin for VEP and Ensembl release 85 [24]. LOFTEE assigns confidence to loss of function annotations based on position of variant in the transcript, proximity to canonical splice sites, and conservation of the putative LOF allele across primates. For our analysis we used default LOFTEE filter setting and only included high confidence predicted LOF variants. A variant was called LOF if it received a high confidence LOF prediction in any Ensembl transcript.

Predicted variant effects were obtained using Annovar v.2014Jul14 [43]. Annovar returns a single prediction for each variant position, collapsing across transcripts and reporting the most damaging variant prediction.

Allele frequencies were obtained from ExAC v0.3.1 and used for comparison to our cohort. [23]

We quantified capture efficiency in this analysis as the percentage of capture target area covered by at least 20 X read depth (denoted C20X). Sequence depth information was obtained on BAMs downloaded from CGhub using GATK ‘DepthOfCoverage’ and the corresponding exon capture bed file to define coverage intervals. Gene level read depth information was obtained from a 5113 BAM files using GATK ‘DepthOfCoverage’ and a RefSeq exon coordinate file obtained from UCSC’s table browser [44, 45]. For the gene level depth analysis, files were downloaded from GDC legacy archive to preserve the original sequence alignment [35].

### Realignment Comparison

To assess the effect of heterogeneous alignment protocols on variant calls, we realigned the raw sequence data for a subset of our cohort. We chose 345 samples to represent a large range of sample preparation variation present in the TCGA BAM files. Reads were stripped from the BAM to generate a FASTQ file using samtools v.0.1.18 bam2fq [46]. The FASTQ was realigned to GRCh37 using BWA MEM v.0.7.12 (with parameters -t 3 -p -M) and duplicates were marked using Picard v.1.131 [47, 48]. From this point the realigned BAM file was processed through the same GATK pipeline described above to produce individual gVCFs. To directly compare the effect of realignment, we generated a VCF for the 345 realigned samples (NewAlign) and for the same 345 samples processed without the realignment step (OldAlign). We were unable to run GATK indel VQSR on a cohort of this size, thus we filtered both VCFs with GATK SNV VQSR TS 99.5 and GATK indel hardfilters (settings QD > 2, FS < 200, ReadPosRankSum > -20). We calculated discordance between alignment pipelines as the percent discordant variant calls: 1- (intersection of variant calls/union of variant calls). Variant calls were matched by position and alternate base, disregarding zygosity.

### WGA Enriched Indels

Indel allele counts were obtained for *n* = 614 WGA and *n* = 9004 DNA samples separately. For each indel site, we obtained a contingency table of the number observed alternate allele counts vs number reference allele counts in DNA vs WGA samples. Reference allele counts were calculated as (2 * the number of samples) - alternate allele count. A one-way Fisher’s exact test was used to define indels with allele counts enriched in WGA samples. A threshold of *p* < 0.063 was used to define WGA enrichment. This cutoff corresponds to the *p* value of a one-way Fisher’s exact test for a singleton present only in WGA samples. Using this method we define *n* = 5654 WGA-enriched and *n* = 34,880 non-enriched indels.

### Homopolymer Indel Analyses

To determine if indels occurred within homopolymer sequences, we obtained the GRCh37 reference sequence +/- 10 base pairs from each indel start position. The only indels considered for homopolymer analysis were those that were single base insertions or deletions or multi base insertions or deletions of the same base. All indels used for homopolymer analysis were < 15 bp in length. An indel was labeled as a homopolymer + indel if a sequential repeat of the inserted/deleted base/s occurred within +/- 1 bp of the indel start position. Using this method we labeled every indel in the pan-cancer VCF as homopolymer +/-. The GC content of the region +/- 10 bp of each indel was additionally determined as number G,C bases/total number of bases.

Homopolymer content by gene was determined using RefSeq coding exon definitions and the GRCh37 reference sequence [45]. For this analysis a homopolymer region was defined as four or more sequential repeats of a single base pair. For each gene, the sequence of all coding exon regions was scanned for homopolymer sequences. Sum totals of number of homopolymers of each type (A,T,C,G) were obtained. G/C and A/T homopolymers were considered together by summing single base homopolymer counts. To compare homopolymer content across genes of different sizes, these counts were divided by the total number of base pairs in the gene’s coding region to obtain the homopolymer count per exonic basepair.

### Chimera Read Analysis

We define large indels as those with an inserted or deleted sequence > = 15 base pairs in length. We identify *n* = 1418 WGA-enriched and *n* = 2301 non-enriched large indels. The inserted or deleted sequence for each indel was aligned to the GRCh37 reference genome using ncbi-blast-2.6.0+ (with parameters -reward 1 -outfmt 6 -num_alignments 1 -max_hsps 3) [28]. For insertions, the match with the highest predicted similarly was retained. For deletions, the best match excluding the actual deleted reference sequence was retained. For all indels with a BLAST hit, the distance between the start position BLAST hit and the indel start position was determined. Indels with BLAST hits > 10 kB away from the indel start position were excluded from this analysis, as MDA chimera artifacts act predominantly within a 10kB proximal region [27].

### Repeated Samples

A subset of individuals in our cohort have multiple germline DNA WXS samples. This cohort of 9618 samples represents 9099 unique individuals; 1012 of the normal WXS samples were obtained from 492 individuals (2–5 samples per individual). The repeated samples all represent germline DNA from the individual, but differ in terms of sample preparation, sequencing, and processing. Percent discordance between repeated samples was calculated as described above. One sample (TCGA-BH-A0BQ) was removed from future analysis due to a high discordance between two high coverage DNA samples. We suspect a sample label mismatch. For association testing, we selected one the sample with the highest coverage that was not whole genome amplified, leaving 9098 samples.

### Indel Filter Methods

To assess different indel filtering methods, indels were extracted from the raw pan-cancer VCF using GATK ‘SelectVariants’. Multialleleic sites containing both SNPs and indels were included in the indel VCF. Four filter methods were tested on the pan-cancer indel VCF: GATK VQSR TS 90.0, TS 95.0, TS 99.0, and GATK Hardfilter. GATK VQSR and Hardfilter filters were applied using the modules ‘ApplyRecalibration’ and ‘VariantFiltration’ respectively (Hardfilter settings QD > 2, FS < 200, ReadPosRankSum > -20). Indels were additionally identified using Varscan v.2.3.9 (with parameters --*p*-value 0.1 --strand-filter 1) on BAMs downloaded directly from CGhub with no preprocessing [49]. Single sample indel VCFs were generated using Varscan for all 9618 samples in our cohort.

### Statistical Methods

To detect contribution of technical factors to LOF variant burden Type II ANOVA was performed using the R package”car” [50]. To determine the percent variance explained by technical factors the sum of squared error for each factor was divided by the total sum of squared error. To create 95% confidence intervals for non-normally distributed data, we used the R package “boot” [51]. The mean for each of 1000 bootstrap samples was calculated and a confidence interval was constructed using the boot.ci function with type set to “basic”.

To detect association between germline gene LOF status and cancer type, we used an ‘one vs. rest’ approach. For each cancer type, a binary (‘dummy’) vector was created indicating whether each individual had the given cancer type (1) or another cancer type (0). For sex specific cancers, only individuals of the same gender were compared. LOF variants with AF < 0.05 were binned by individual by gene to generate on individual LOF variant count for each gene. Genes were only included in our analysis if at least two individuals in the cohort had germline LOF variants in the gene. For each cancer type and each gene we used a logistic regression to test association between germline LOF variant burden and cancer type. Our regression model took the form: glm(cancer type indicator ~ variant burden + race + age). To discover significant gene-cancer type associations we obtained the *p* value of the β coefficient for the variant burden term and used a Bonferroni cutoff of 1.61 X 10^-7^ to account for multiple testing (31 cancer types x ~10,000 genes).

## Additional files

Additional file 1: Supplementary Figures and small Tables. (PDF 4248 kb)

Additional file 2: Technical covariates for 9618 samples in pan-cancer cohort. (XLSX 600 kb)

Additional file 3: TCGA samples with discrepancy between self-report and PCA predicted ancestry. (XLS 60 kb)

Additional file 4: Significant genes from logistic regression analysis. (XLSX 94 kb)

## Abbreviations

ANOVA: Analysis of variance
BAM: Binary alignment/map
BLAST: Basic local alignment search tool
bMMRD: Bialleleic mismatch repair deficiency
BWA: Burrows wheeler aligner
CGhub: Cancer genomics hub
COAD: Colon adenocarcinoma
dbGAP: Database of genotypes and phenotypes
ExAC: Exome Aggregation Consortium
FS: Fisher strand
GATK: Genome analysis toolkit
GDC: Genomic data commons
gnomAD: Genome aggregation database
Indel: Insertion/deletion
LAML: Acute Myeloid Leukemia
LOF: Loss of function
LOFTEE: Loss of Function Transcript Effect Estimator
MDA: Multiple displacement amplification
OV: Ovarian cancer
PCA: Principal component analysis
QD: Quality by depth
READ: Rectum adenocarcinoma
RefSeq: Reference sequence
SNV: Single nucleotide variant
TCGA: The Cancer Genome Atlas
TCGT: Testicular germ cell tumors
TS: Truth sensitivity
UVM: Uveal melanoma
VCF: Variant call format
VEP: Variant effect predictor
VQSR: Variant Quality Score Recalibration
WGA: Whole genome amplification
WXS: Whole exome sequencing
