# Supplementary material for: Pan-cancer analysis reveals technical artifacts in TCGA germline variant calls
Source: BMC Genomics. 2017 Jun 12;18:458. doi: 10.1186/s12864-017-3770-y (PMC5467262; doi:10.1186/s12864-017-3770-y)
Supplement: Supplementary file 1 — Supplementary Figures and small Tables. (PDF 4248 kb) [file 12864_2017_3770_MOESM1_ESM.pdf]

S. Figure 1

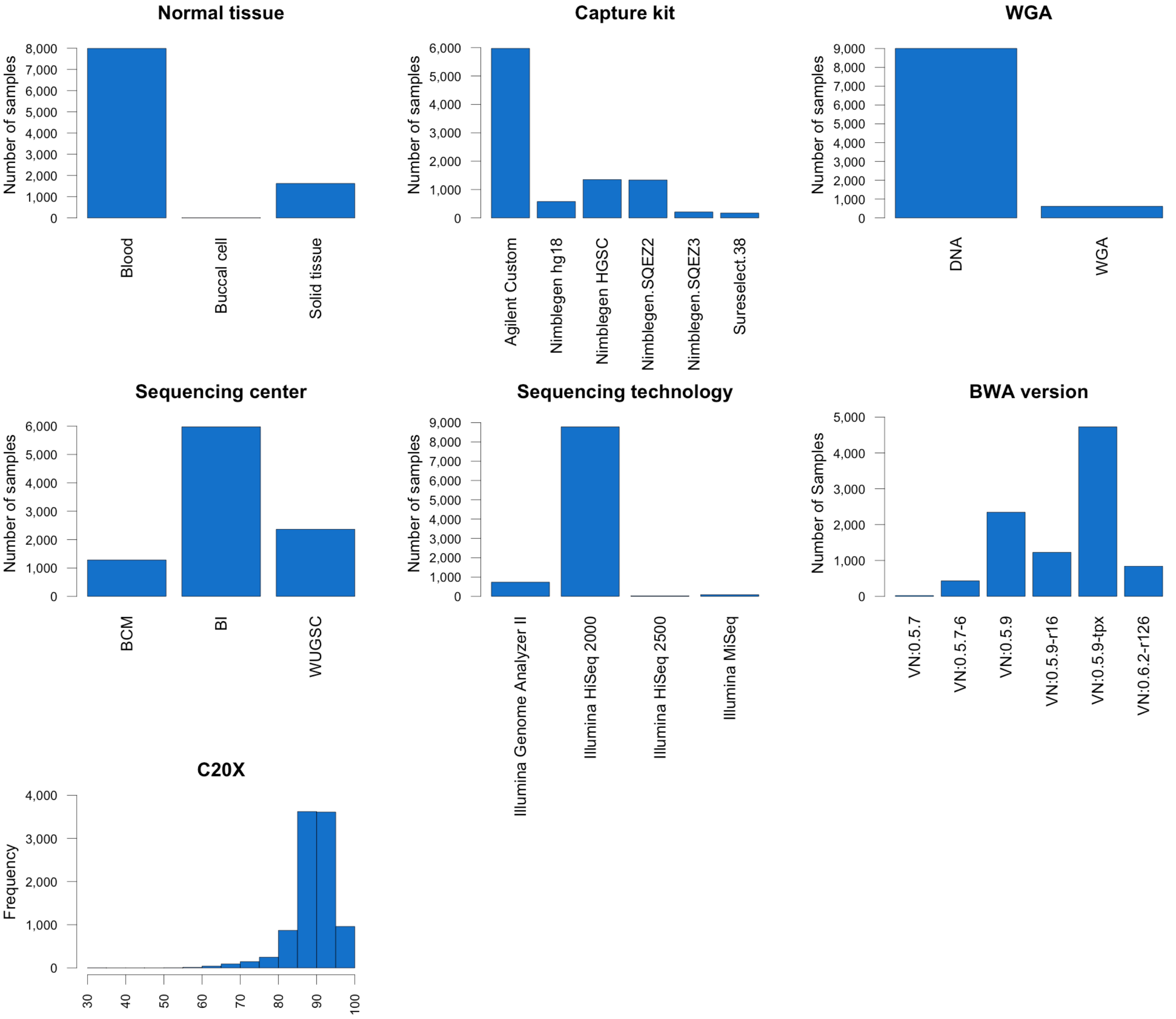

S1 Fig. Distribution of technical covariates for the pan-cancer cohort
The distribution of the seven identified technical covariates for *n*=9618 TCGA WXS samples. Capture efficiency is measured as percentage of capture target area covered by at least 20 X read depth (denoted C20X)

S. Figure 2

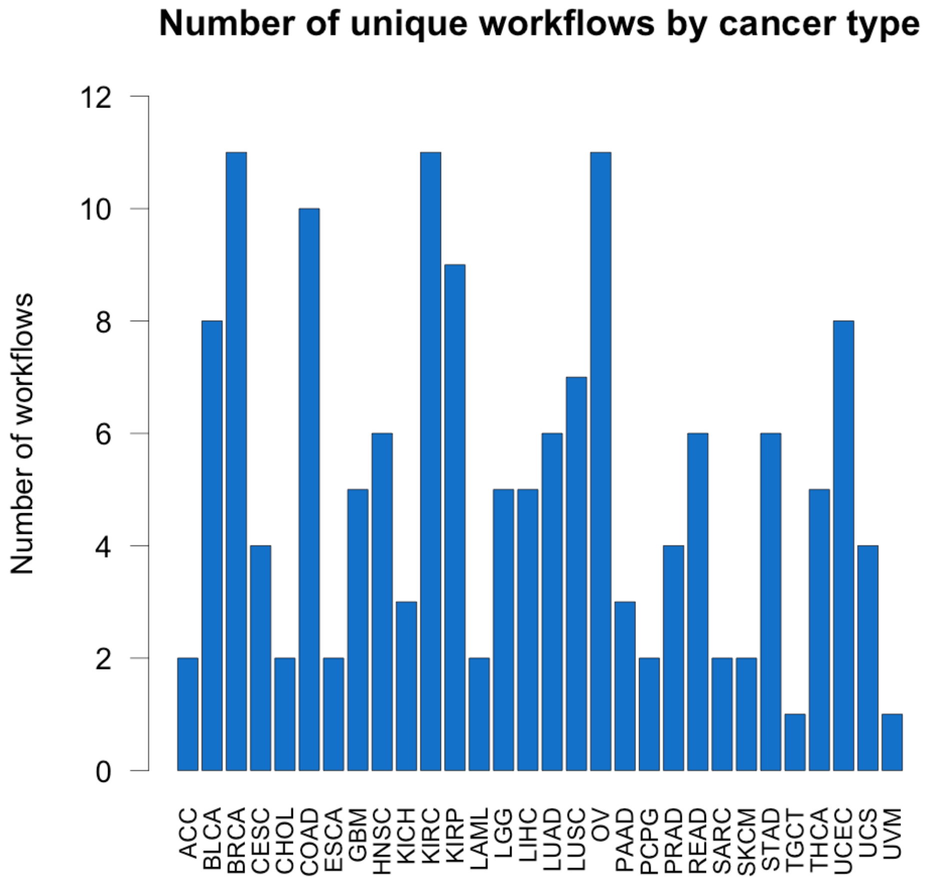

S2 Fig. Number of processing workflows used to generate TCGA WXS data

The number of unique combinations of six technical factors (sequencing center, normal tissue, WGA, BWA version, capture kit, and sequencing technology) per cancer type

S. Figure 3

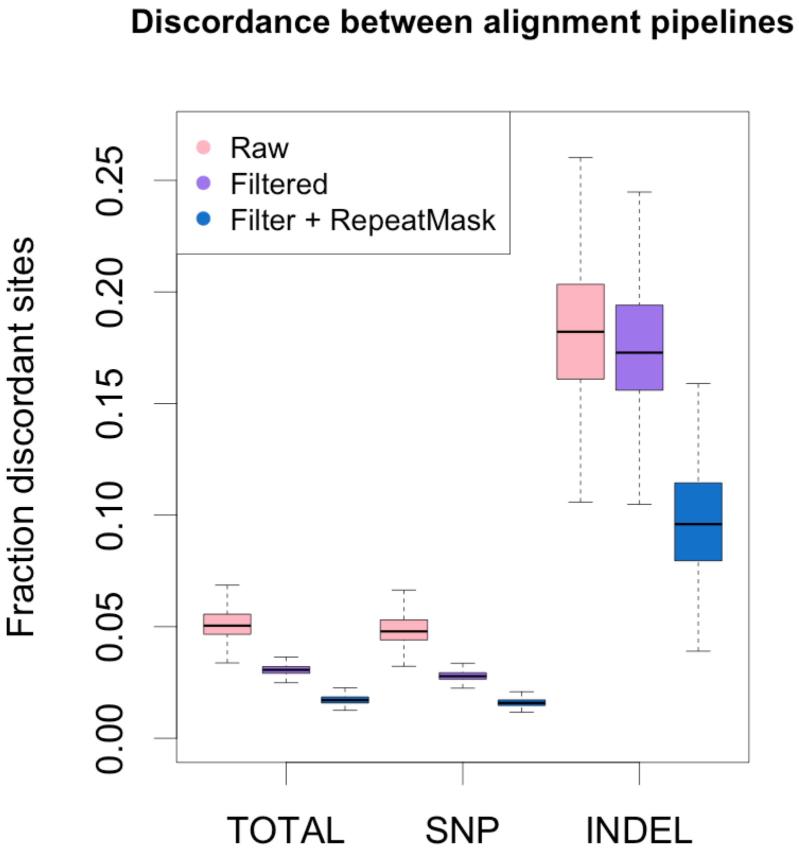

S3 Fig. Variant call discordance between NewAlign and OldAlign samples (*n*=345)

For filtered condition SNPs were filtered using GATK VQSR TS 99.5 and indels using GATK hardfilter. For filtered + RepeatMask condition variants in UCSC tracks RepeatMasker and Segmental Dups were excluded[42].

S. Figure 4

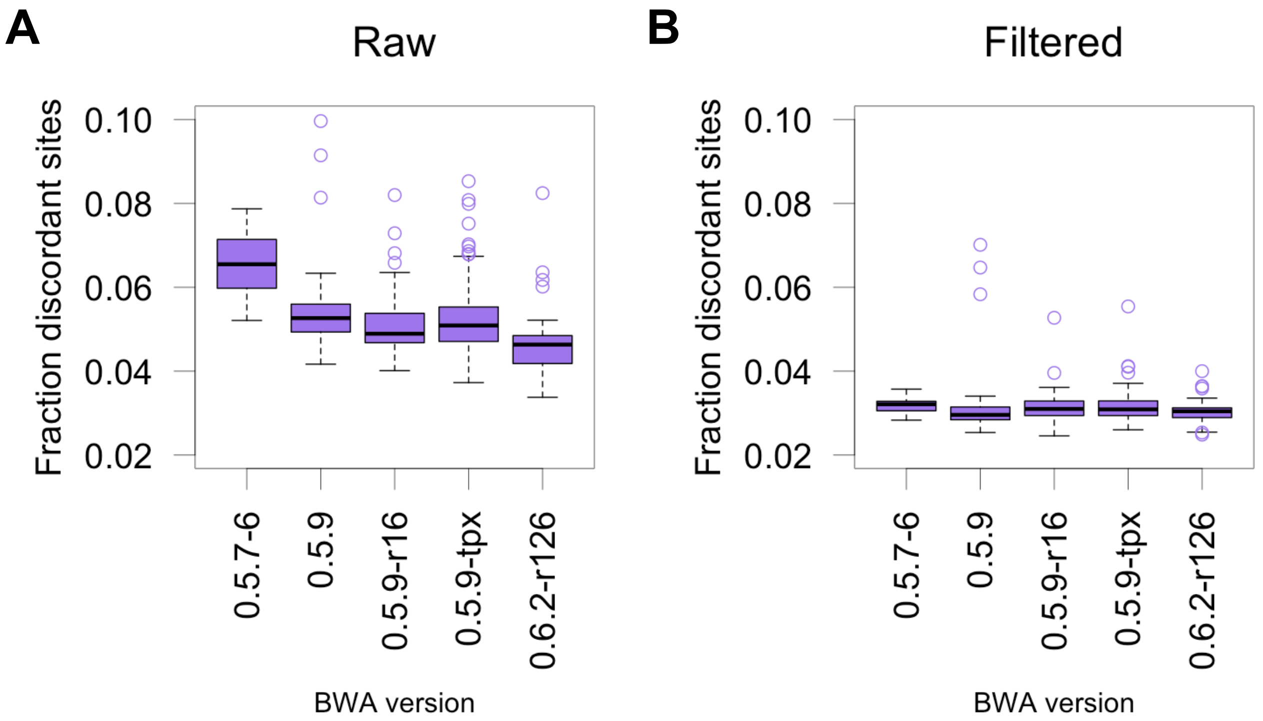

S4 Fig. Discordance with BAM realignment plotted by BWA version used to generate BAM file (A) Raw VCF discordance between NewAlign and OldAlign samples plotted by BWA version. (B) Filtered VCF discordance between NewAlign and OldAlign samples plotted by BWA version. SNVs were filtered at GATK VQSR TS 99.5, indels with GATK Hardfilter.

S. Figure 5

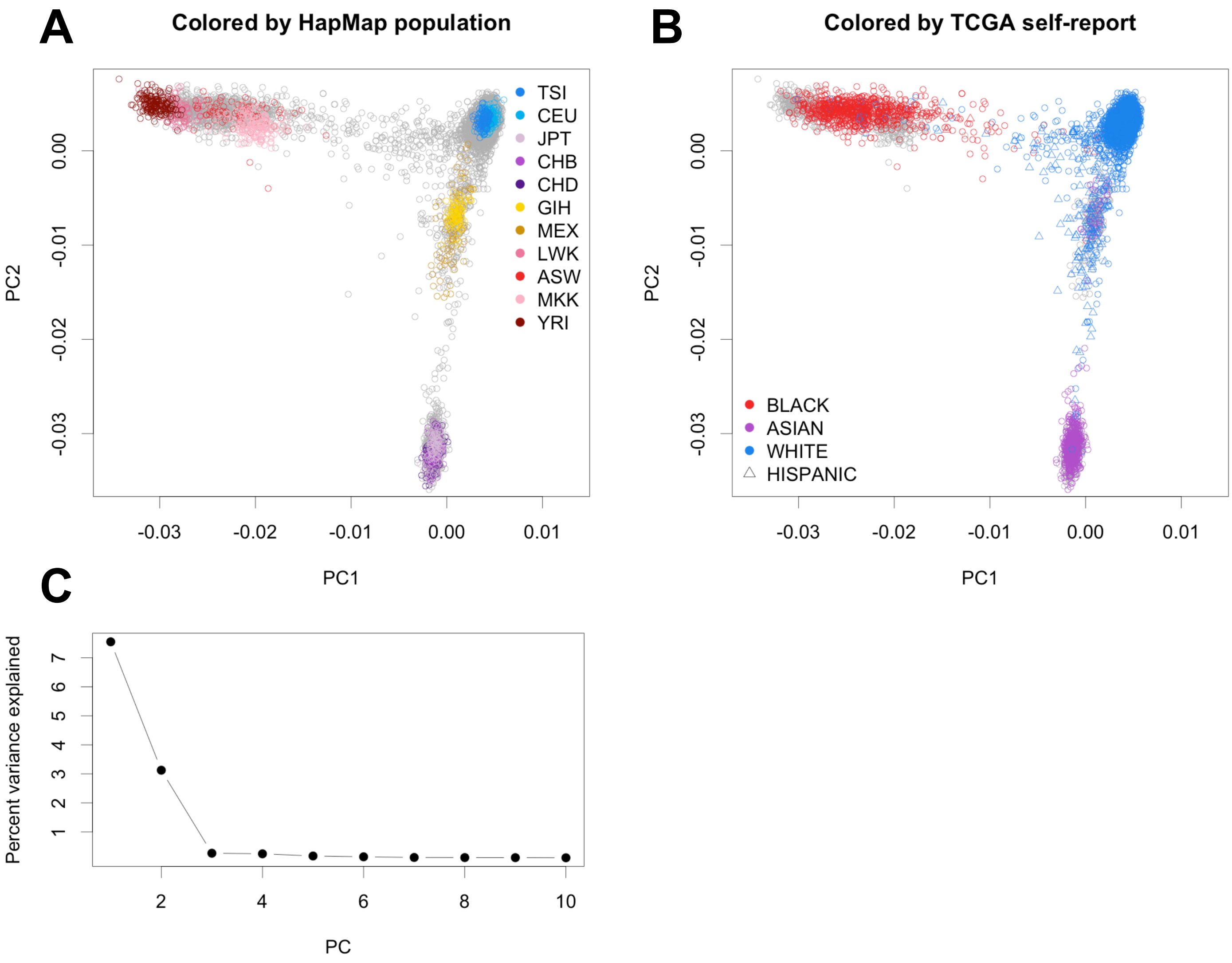

S5 Fig. PCA of common variants from pan-cancer VCF

(A) Principal components (PC) 1 and 2 from joint pan-cancer and HapMap analysis, HapMap samples are colored by population. HapMap abbreviations: ASW, African ancestry in Southwest USA; CEU, Utah residents with Northern and Western European ancestry from the CEPH collection; CHB, Han Chinese in Beijing, China; CHD, Chinese in Metropolitan Denver, Colorado; GIH, Gujarati Indians in Houston, Texas; JPT, Japanese in Tokyo, Japan; LWK, Luhya in Webuye, Kenya; MXL, Mexican ancestry in Los Angeles, California; MKK, Maasai in Kinyawa, Kenya; TSI, Toscani in Italia; YRI, Yoruba in Ibadan, Nigeria. (B) Same data as A, TCGA samples are colored by self-report ancestry. (C) Percent total variance explained by the top 10 PCs.

S. Figure 6

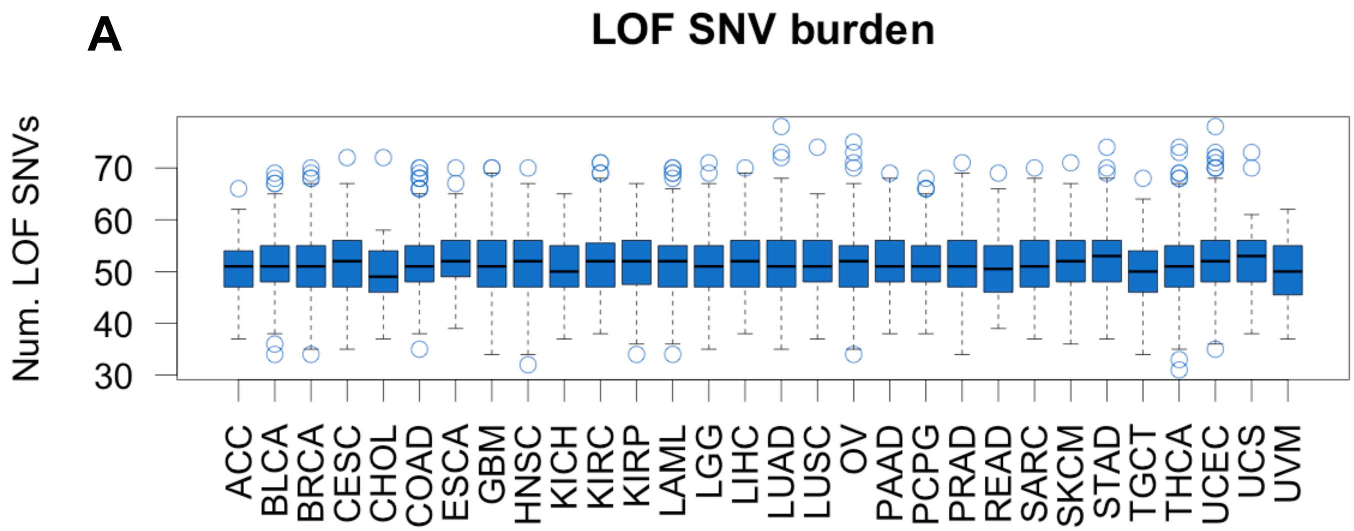

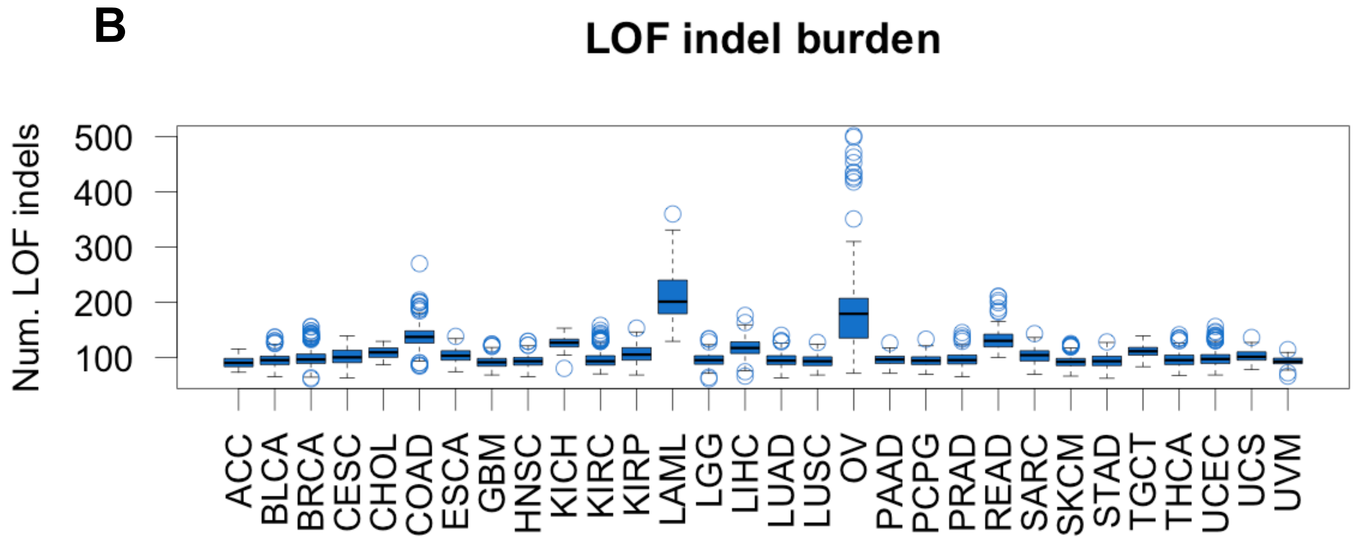

S6 Fig. LOF variant burden split by variant type

(A) Individual LOF SNV burden plotted by cancer type. (B) Individual LOF indel burden plotted by cancer type

S. Figure 7

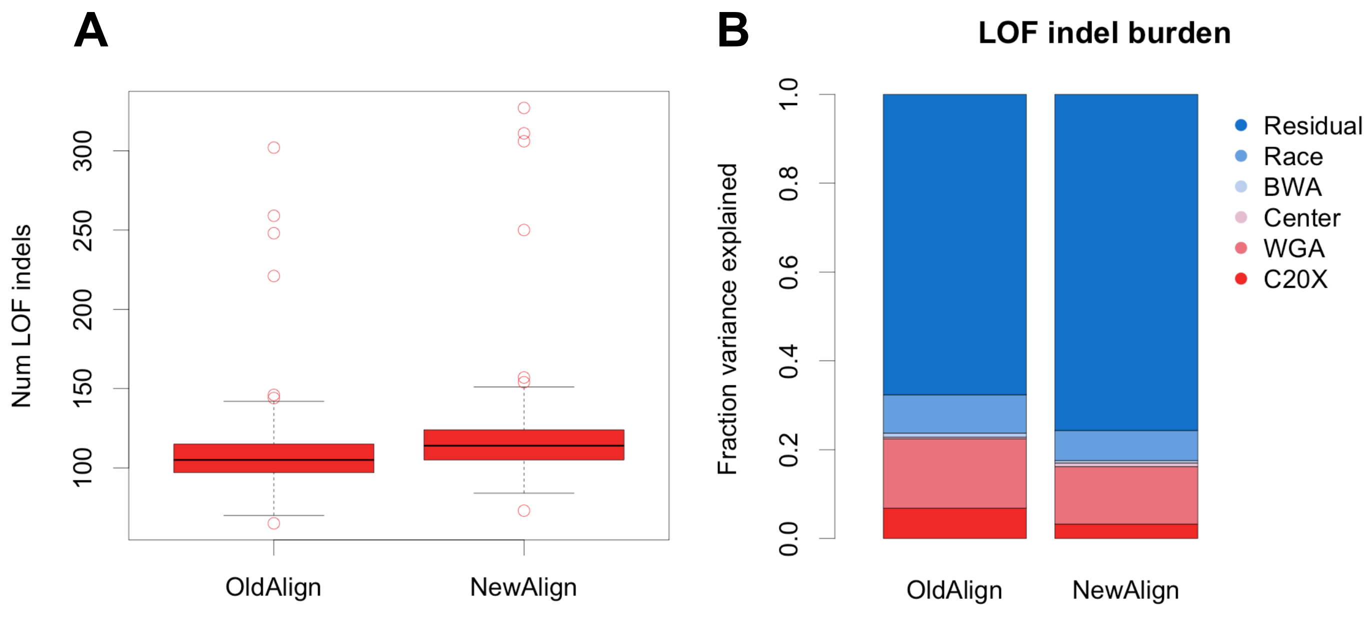

S7 Fig. Variance in LOF indel burden explained by technical factors in NewAlign cohort

(A) Number of LOF indels per individual in NewAlign and OldAlign pipelines. There were a median 8 more LOF indels in the NewAlign pipeline. Overall individual LOF indel burden was highly correlated between pipelines (Pearson $R^2 = 0.947$). (B) Percent of variation in individual LOF indel burden explained by technical covariates as assessed by ANOVA.

S. Figure 8

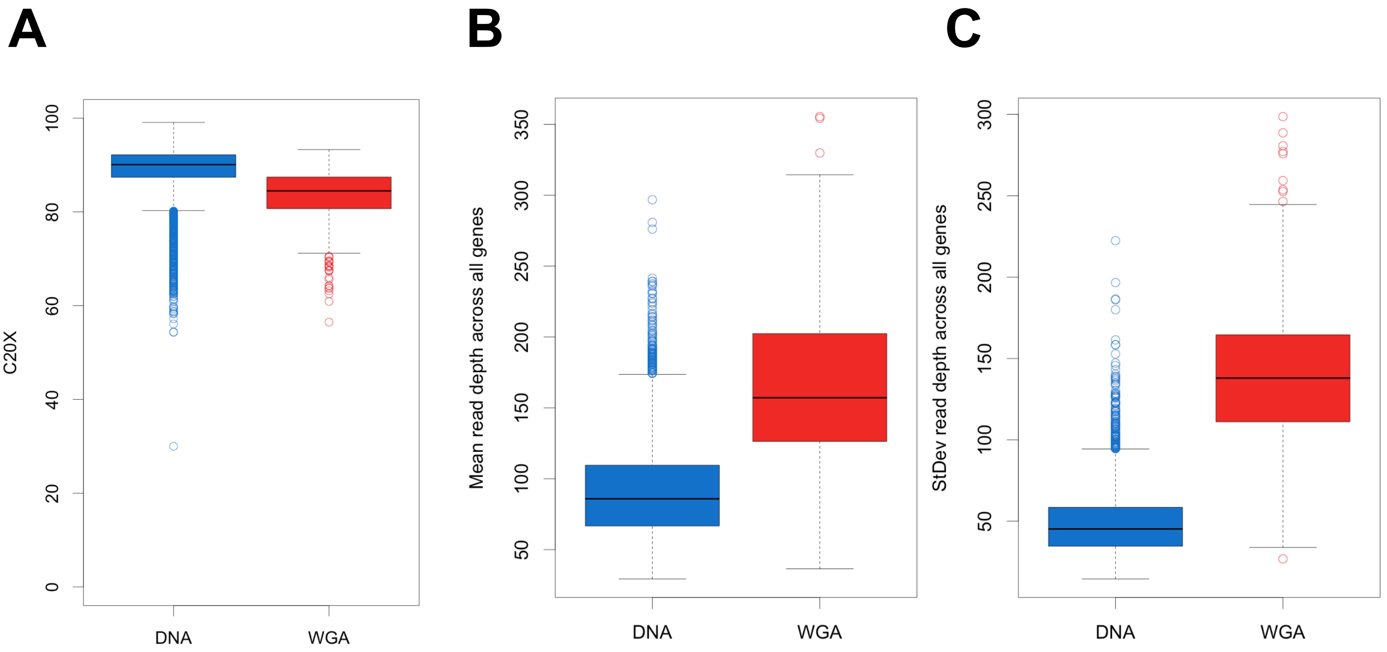

S8 Fig. Variability in coverage and read depth in WGA samples

(A) C20X plotted by WGA status. (B) Mean read depth per individual across 16,824 genes for *n*=446 WGA samples and *n*=4,667 DNA samples. (C) Standard deviation in read depth per individual across 16,824 genes.

S. Figure 9

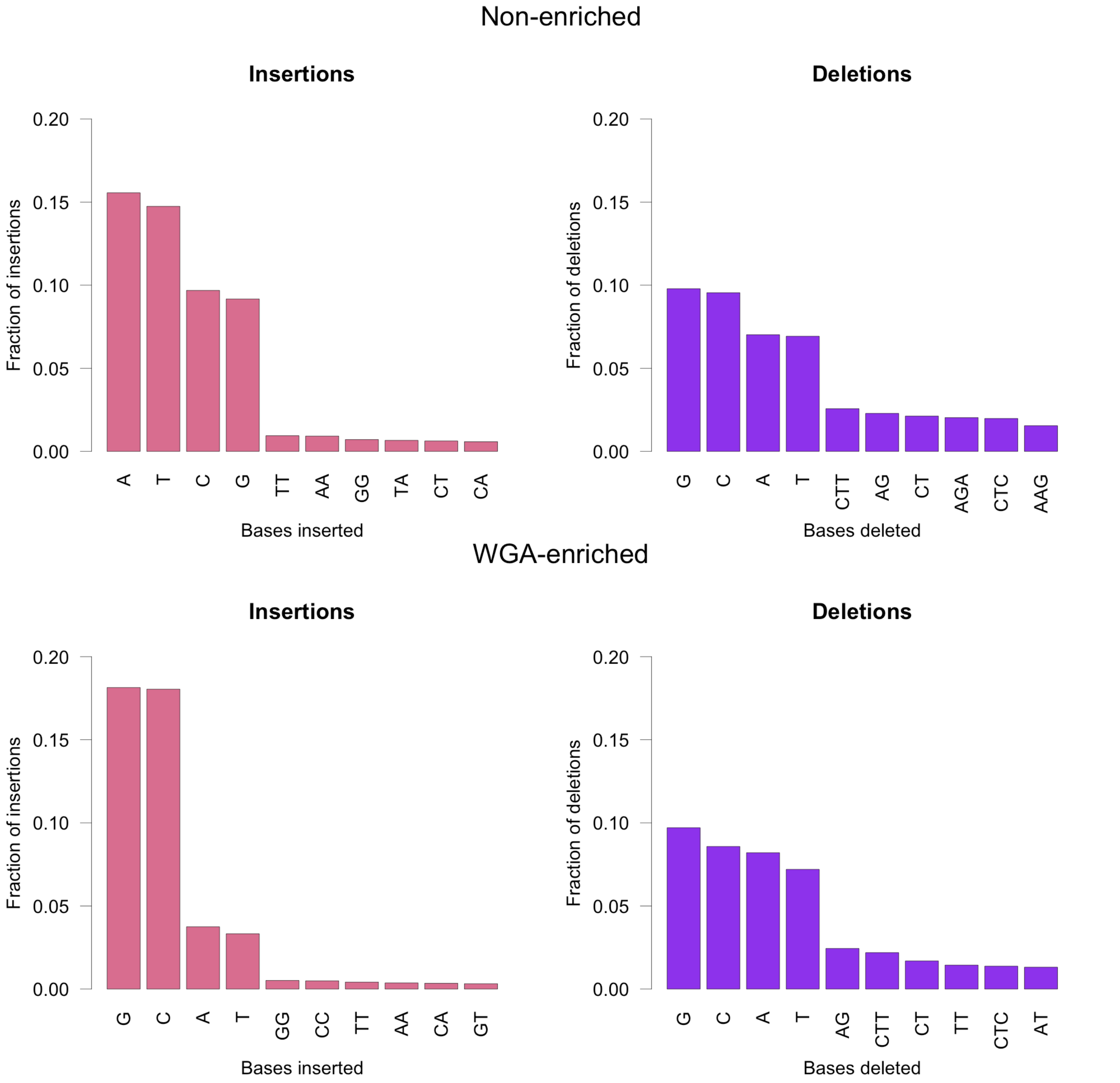

S9 Fig. Frequently inserted and deleted bases of WGA-enriched and non-enriched indels

The ten most frequent inserted or deleted base pairs for WGA-enriched and non-enriched indels. The height of the bar indicates frequency of each insertion or deletion relative to all insertions or deletions in that indel set.

S. Figure 10

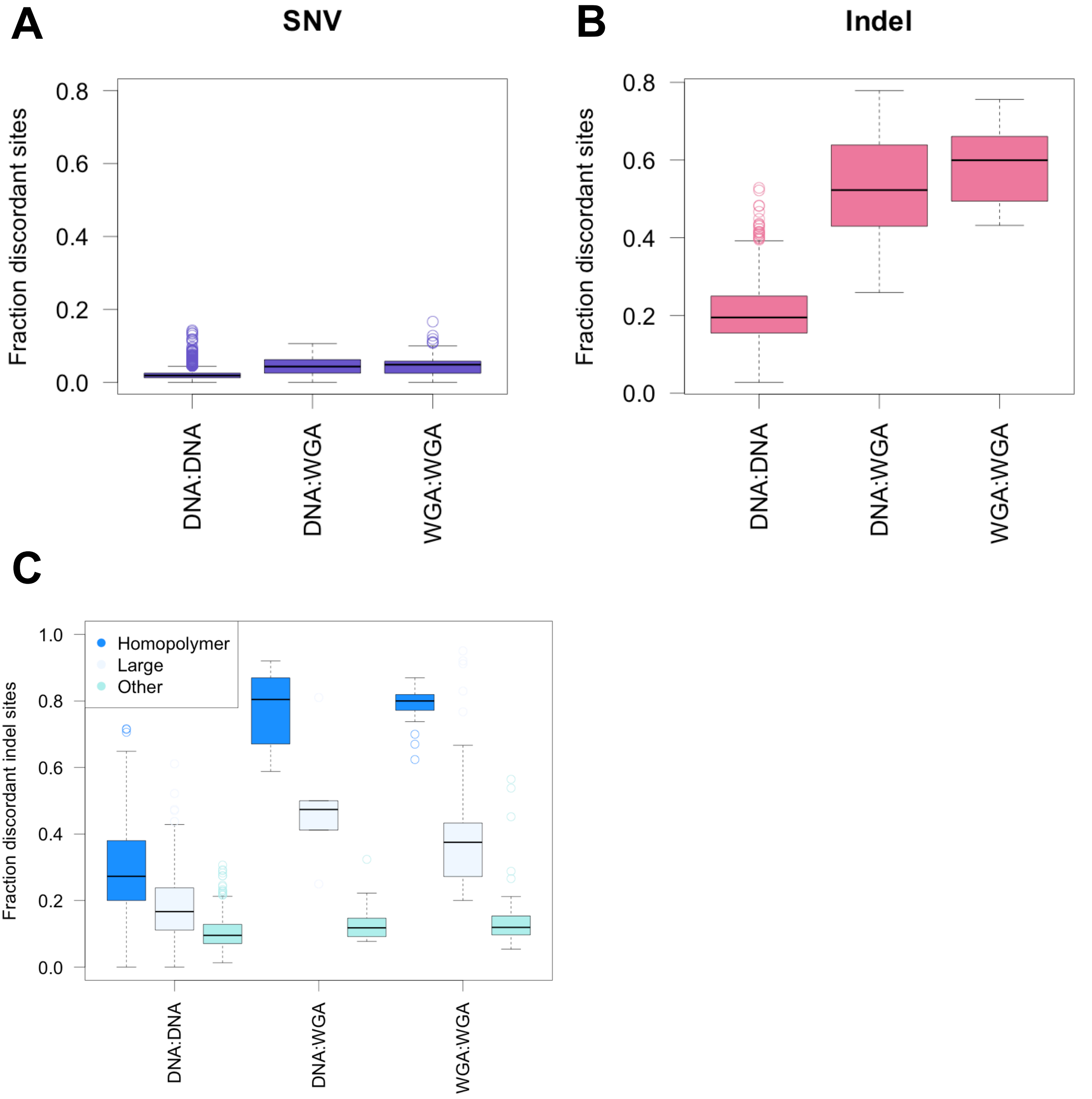

S10 Fig. Discordance between repeated WXS samples with and without WGA

Discordance between repeated samples of *n*=492 individuals plotted by WGA status. DNA:DNA = all samples are DNA, WGA:DNA = at least one sample is WGA, WGA:WGA= all samples are WGA. Discordance was calculated separately for SNVs (A) and indels(B). (C) Indel discordance on the same samples calculated separately for hompolymer + indels, indels 15 base pairs or longer, and other indels.

S. Figure 11

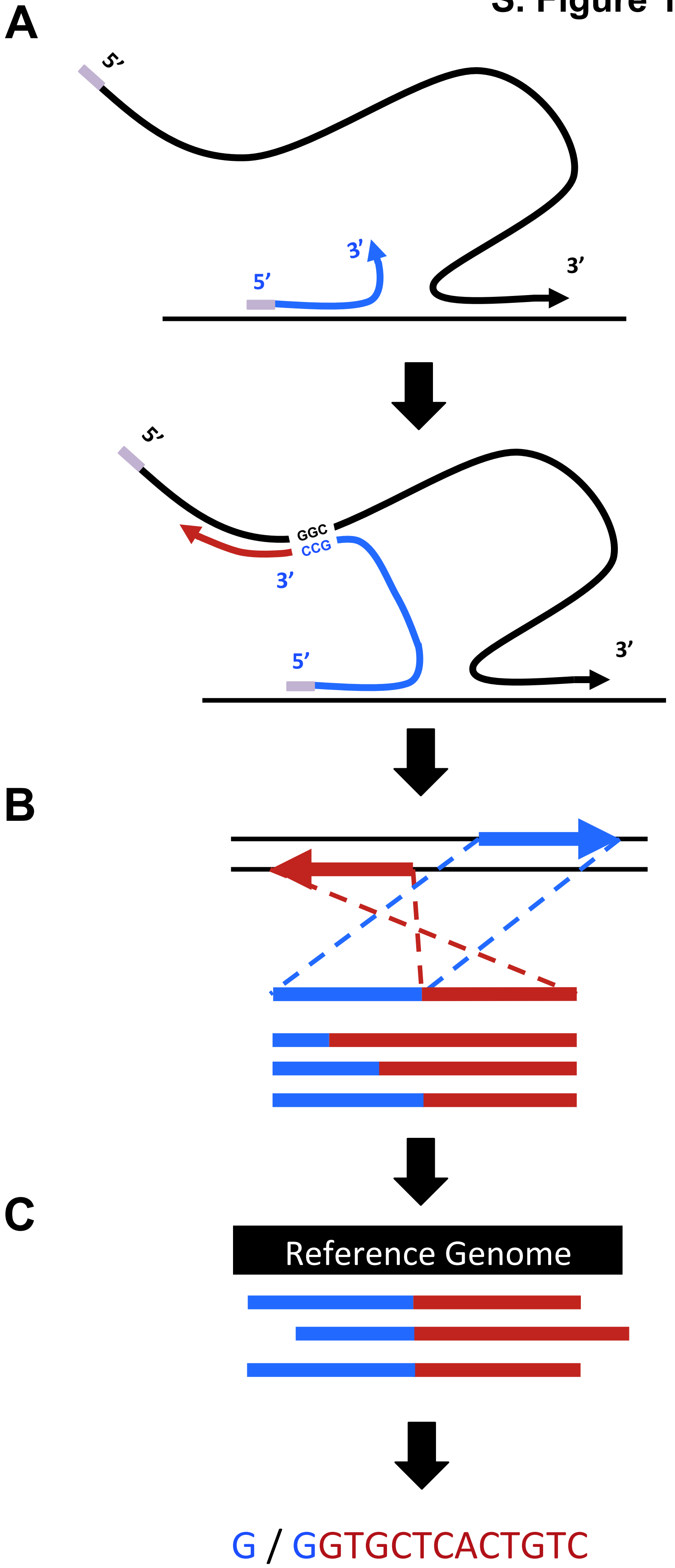

S11 Fig. Proposed mechanism of artifactual indel generation by MDA chimera reads

(A) Branching during MDA creates free 3' termini that can anneal to proximal 5' strands with complementary sequence, generating chimera events[27]. These events most frequently occur within a 10 kB window. (B) Chimera events manifest in sequence data as reads containing sequence from two noncontinuous regions of the reference genome. Here we demonstrate a chimera read formed by an inverted rearrangement with a deletion. (C) Chimeric reads can be discarded during multiple stages of variant calling, including initial alignment of reads to the genome, GATK's indel realignment step, or GATK's 'HaplotypeCaller' pairHMM realignment[15]. We observe that chimeric reads that persist to the final stages of variant calling resemble insertions of varying sizes.

S. Figure 12

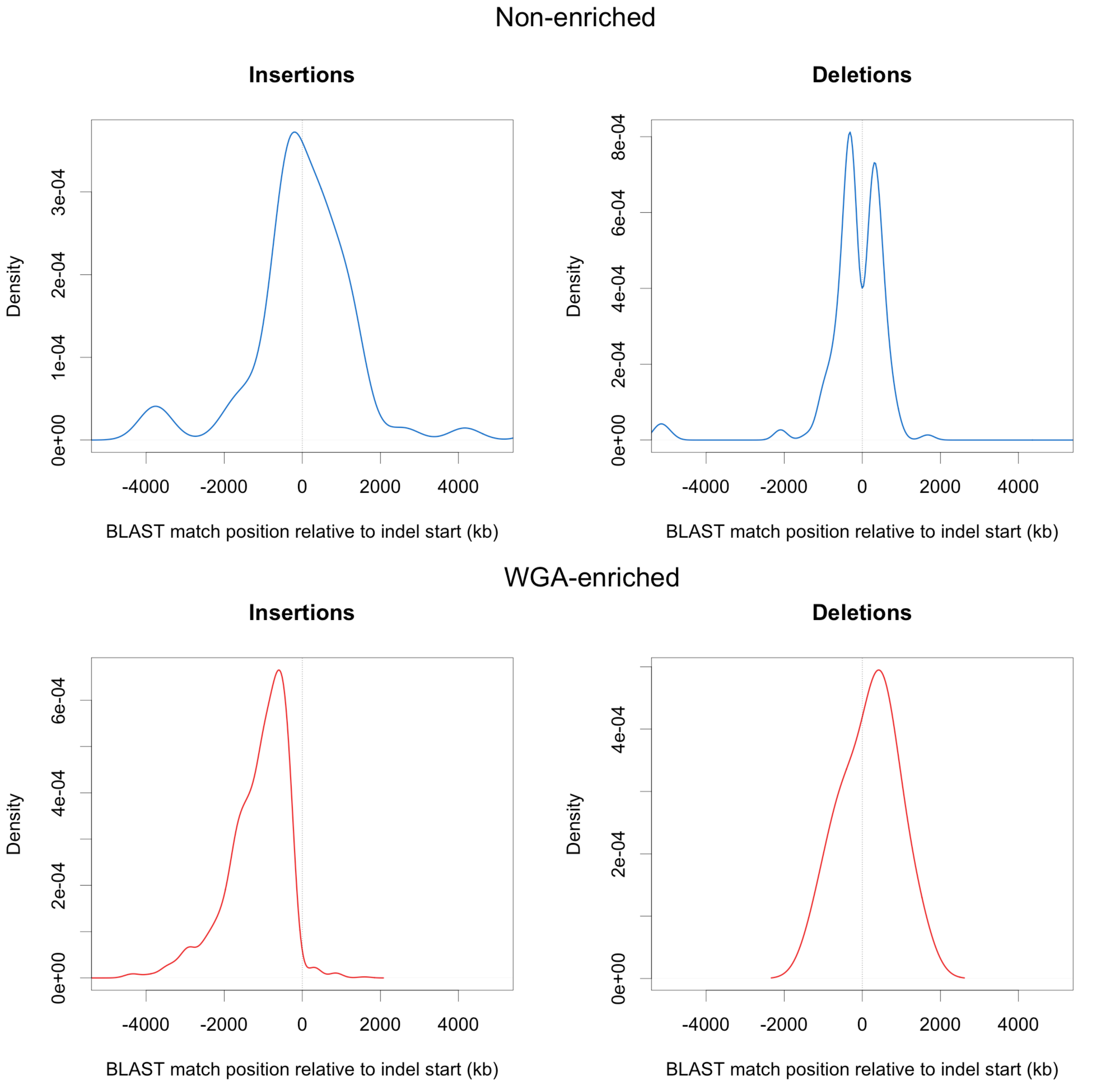

S12 Fig. Distribution of indel sequence BLAST hits relative to indel start position

For WGA-enriched and non-enriched large insertions and deletions with BLAST matches, the location of BLAST matches are shown. Indel start position is 0, *n*=1,113 WGA-enriched insertions, *n*=11 WGA-enriched deletions, *n*=69 Non-enriched insertions, *n*=175 Non-enriched deletions).

S. Figure 13

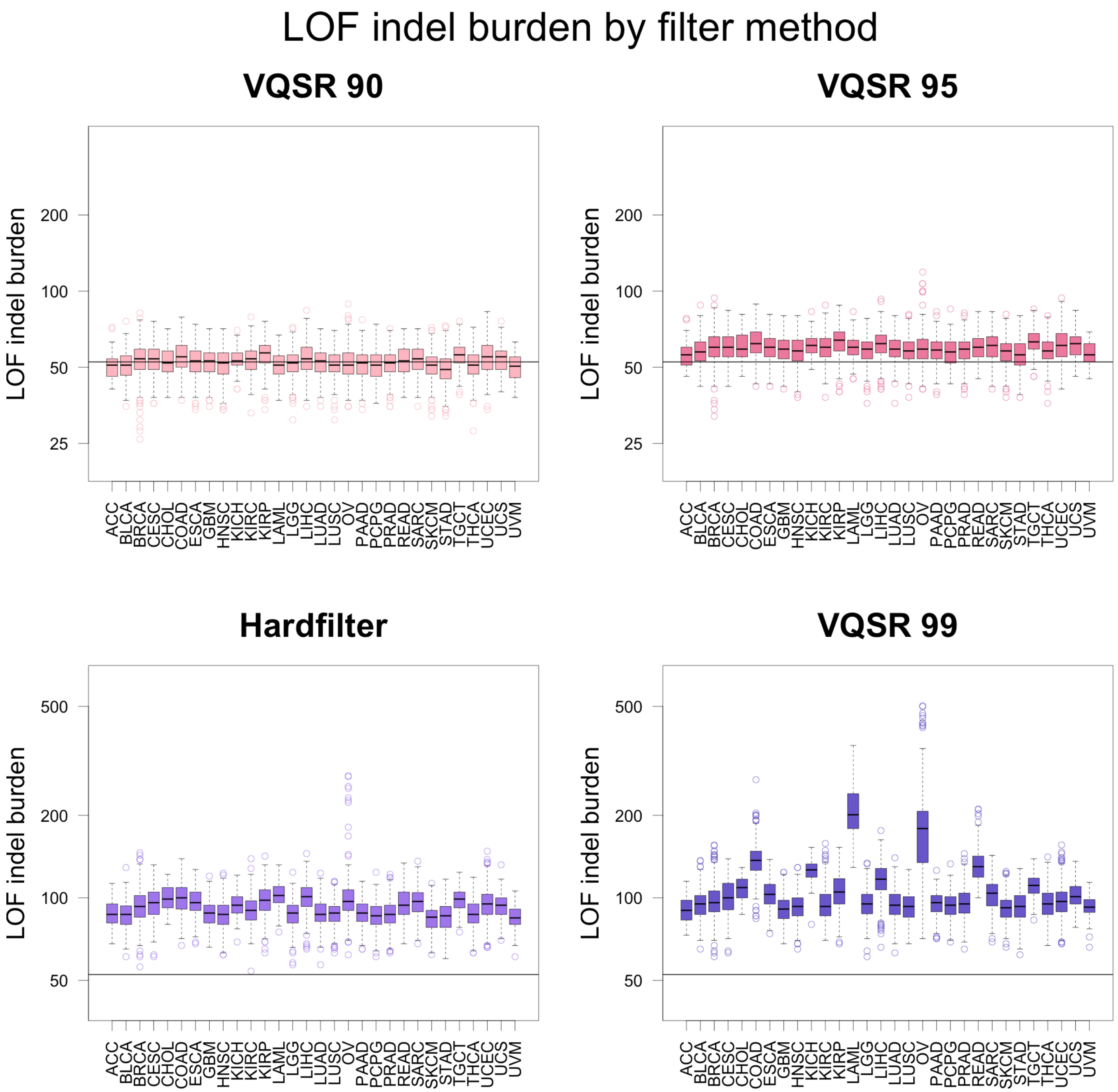

S13 Fig. Individual LOF indel burden by cancer type for all indel filtering methods tested

The black line represents the median LOF indel burden in the most stringent filter condition (VQSR 90) for comparison.

S. Figure 14

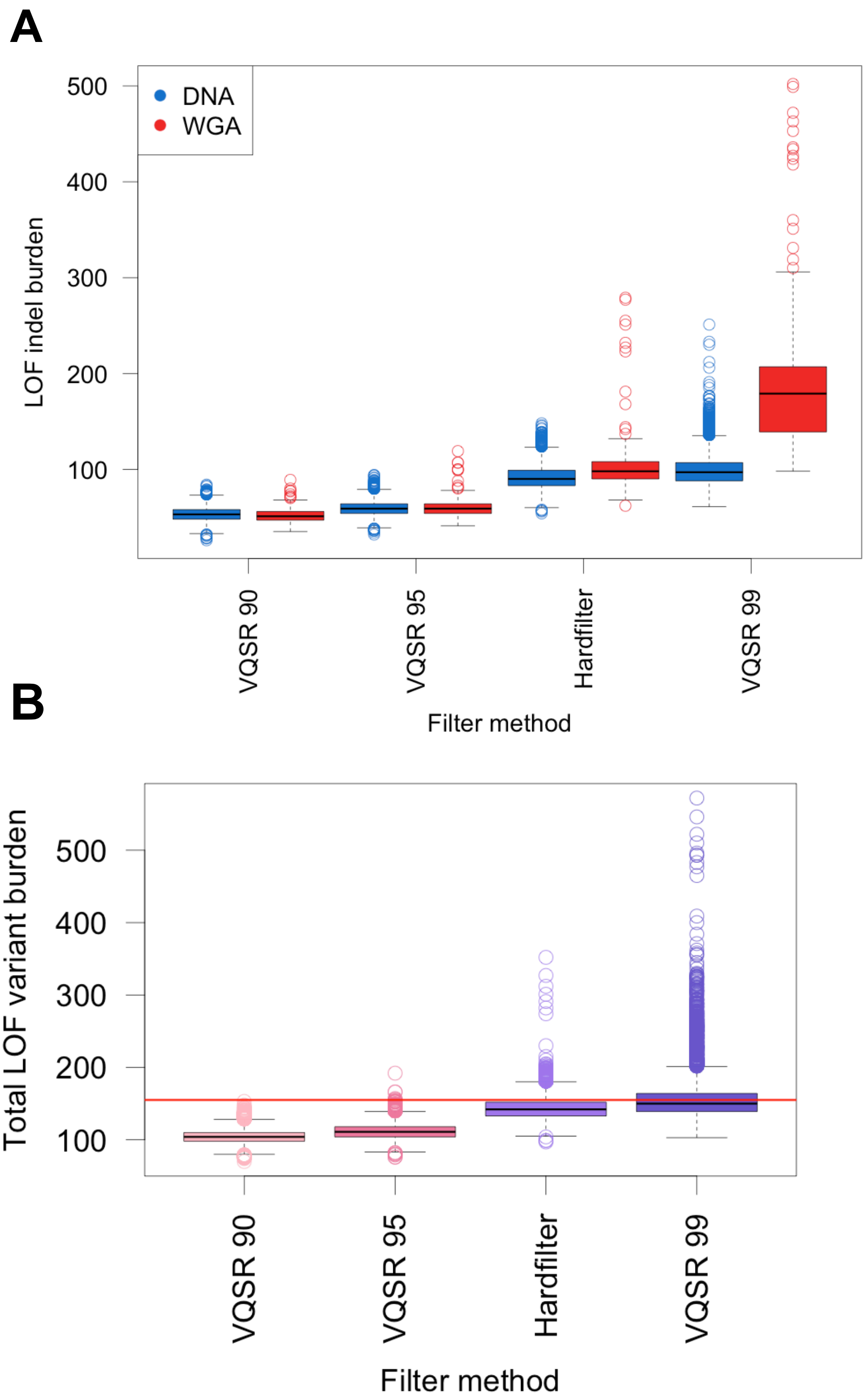

S14 Fig. LOF indel burden plotted by WGA status and total LOF burden for indel filter methods.

(A) Individual LOF indel burden of WGA and DNA samples for each filter. (B) LOF variant count includes both SNV and indels. The red line indicates expected LOF burden from ExAC (155).

S. Figure 15

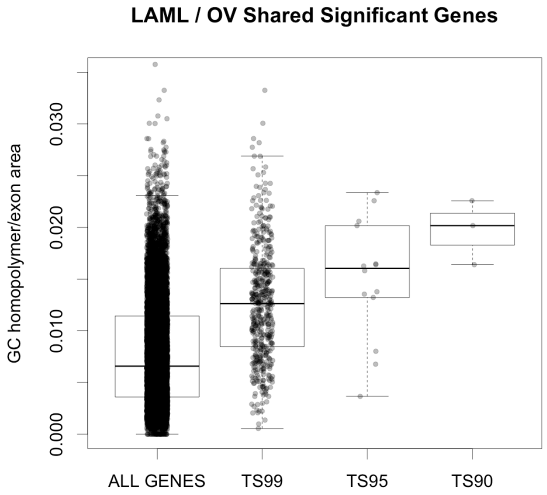

S15 Fig. G/C homopolymer content of significant genes shared between OV and LAML

The number of G/C hompolymer regions normalized by coding exon length in base pairs plotted for all genes and for genes that were significant $p < 1.61 \times 10^{-7}$ by logistic regression for both OV and LAML. Significant genes shared between OV and LAML under three different indel filter conditions (VQSR TS99, TS95, TS90) are plotted for comparison.

S. Figure 16

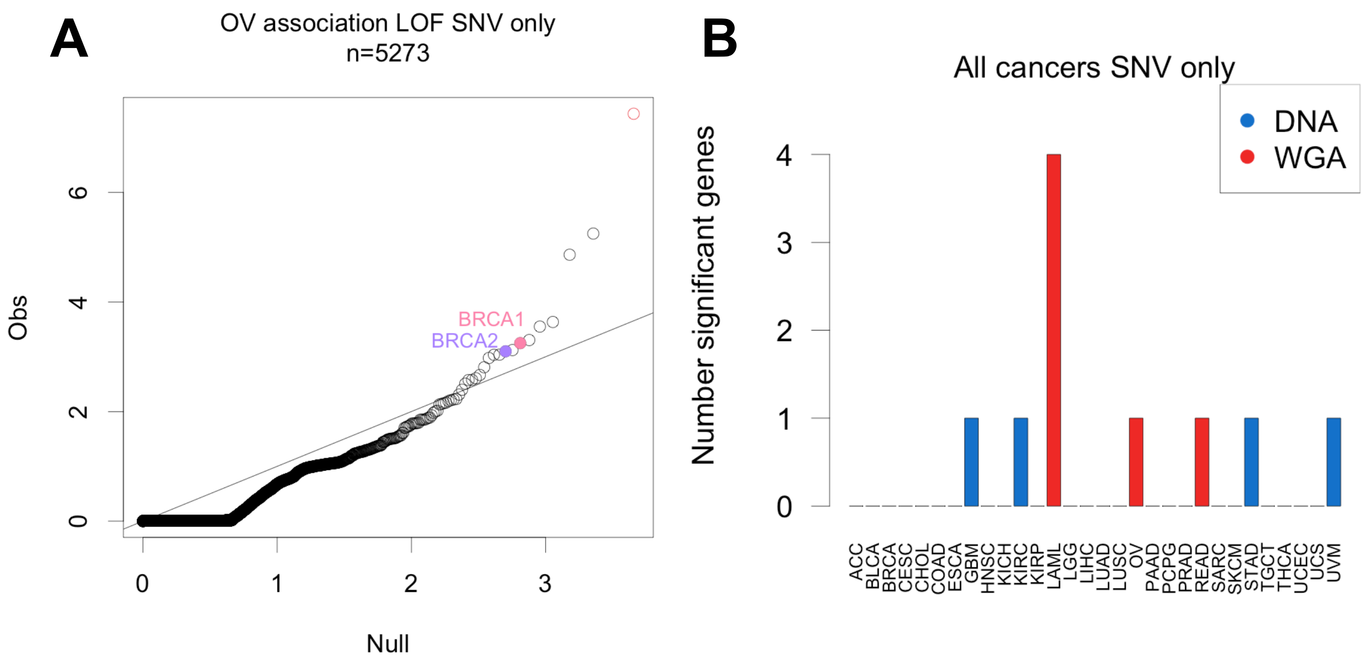

S16 Fig. Logistic regression analysis using only LOF SNVs

(A) Quantile-quantile plot from logistic regression association testing between germline LOF SNV burden and OV. *n*=number of genes tested. Red line indicates significant cutoff and red points indicate associations significant $p < 1.61 \times 10^{-7}$. *BRCA1*/2 associations highlighted. (B) Number of genes significant $p < 1.61 \times 10^{-7}$ by logistic regression for all cancer types. Color indicates cancer types containing WGA samples.

S. Table 1

**ACC**	Adrenocortical carcinoma	89	**LUAD**	Lung adenocarcinoma	575
**BLCA**	Bladder Urothelial Carcinoma	416	**LUSC**	Lung squamous cell carcinoma	327
**BRCA**	Breast invasive carcinoma	849	**OV**	Ovarian serous cystadenocarcinoma	399
**CESC**	Cervical squamous cell carcinoma and endocervical adenocarcinoma	308	**PAAD**	Pancreatic adenocarcinoma	188
**CHOL**	Cholangiocarcinoma	49	**PCPG**	Pheochromocytoma and Paraganglioma	182
**COAD**	Colon adenocarcinoma	325	**PRAD**	Prostate adenocarcinoma	510
**ESCA**	Esophageal carcinoma	190	**READ**	Rectum adenocarcinoma	114
**GBM**	Glioblastoma multiforme	315	**SARC**	Sarcoma	259
**HNSC**	Head and Neck squamous cell carcinoma	585	**SKCM**	Skin Cutaneous Melanoma	472
**KICH**	Kidney Chromophobe	66	**STAD**	Stomach adenocarcinoma	485
**KIRC**	Kidney renal clear cell carcinoma	275	**TGCT**	Testicular Germ Cell Tumors	149
**KIRP**	Kidney renal papillary cell carcinoma	319	**THCA**	Thyroid carcinoma	529
**LAML**	Acute Myeloid Leukemia	123	**UCEC**	Uterine Corpus Endometrial Carcinoma	487
**LGG**	Brain Lower Grade Glioma	516	**UCS**	Uterine Carcinosarcoma	57
**LIHC**	Liver hepatocellular carcinoma	380	**UVM**	Uveal Melanoma	80

S1 Table. Number of samples of each cancer type in the pan-cancer cohort

S. Table 2

Capture Kit	Size (MB)	Fraction Overlap With Gencode Exons	Number Samples
Agilent Custom	33	0.993	5793
Nimblegen SQEZ v2	36	0.996	1337
Nimblegen hg18	36	0.992	577
Nimblegen HGSC	37	0.997	1350
Nimblegen SQEZ v3	39	0.999	210
SureSelect 38	64	0.982	171
Intersection	27	0.977	

S2 Table. Size and overlap with Gencode exons for the six capture kits used to collect TCGA normal DNA samples.

S. Table 4

K-means Cluster	ASW	LWK	MKK	YRI	CEU	TSI	GIH	MEX	CHB
1	83	90	171	167	0	0	0	0	0
2	0	0	0	0	165	88	0	5	0
3	0	0	0	0	0	0	88	72	0
4	0	0	0	0	0	0	0	0	84

S4 Table. K-means cluster membership of HapMap samples from PCA

S. Table 5

K-means Cluster	Black	White	Hispanic	Asian	NA
1	777	25	11	0	103
2	22	6611	129	9	963
3	6	76	152	39	65
4	0	11	6	536	50

S5 Table. K-means cluster membership of TCGA samples from PCA

S. Table 7

	Insertions	Deletions
WGA-enriched	Mean 0.628 (95% C.I. 0.624 - 0.633)	Mean 0.510 (95% C.I. 0.502 - 0.517)
Non-enriched	Mean 0.539 (95% C.I. 0.536 - 0.542)	Mean 0.515 (95% C.I. 0.514 - 0.518)

C.I. Confidence interval for mean estimate derived from 1,000 bootstrap samples

S7 Table. Mean GC content of the sequence surrounding WGA-enriched and non-enriched indels

S. Table 8

	Hompolymer -	Hompolymer +
DNA	Mean 0.0038 (95% C.I. 0.0034 - 0.0043)	Mean 0.0028 (95% C.I. 0.0019 - 0.0036)
WGA	Mean 0.0160 (95% C.I. 0.0141 - 0.0180)	Mean 0.0236 (95% C.I. 0.0213 - 0.0257)

C.I. Confidence interval for mean estimate derived from 1,000 bootstrap samples

S8 Table. Mean allele frequency of indels in homopolymer regions in DNA and WGA samples

S. Table 9

	Num. Insertions	% Insertions with BLAST hit	Num. Deletions	% Deletions with BLAST hit
WGA-enriched	1,310	86.49	108	10.18
Non-enriched	634	10.88	1,667	10.49

Large indels are indels >= 15 base pairs. BLAST hits are defined as a BLAST match +/- 10 kB from the indel start position

S9 Table. Total number of large indels and fraction of large indels with a BLAST hit in WGA-enriched and non-enriched indel sets

S. Table 10

GATK Indel VQSR TS 90.0				
	Sum Sq	Df	F value	Pr(>F)
C20X	1.31E+04	1	323.8629669	4.52E-71
WGA	2.39E+00	1	0.05899371	8.08E-01
Center	1.49E+03	2	18.33944372	1.13E-08
BWA	1.19E+03	5	5.86624688	2.04E-05
Race	5.60E+04	5	276.5583032	1.43E-274
Residuals	3.39E+05	8363		
GATK Indel VQSR TS 95.0				
	Sum Sq	Df	F value	Pr(>F)
C20X	15219.389	1	327.944016	6.31E-72
WGA	1361.419	1	29.335557	6.26E-08
Center	3258.38	2	35.105429	6.57E-16
BWA	1507.617	5	6.497157	4.89E-06
Race	68648.485	5	295.844466	2.06E-292
Residuals	388114.262	8363		
GATK Hardfilter				
	Sum Sq	Df	F value	Pr(>F)
C20X	50615.091	1	419.836036	4.45E-91
WGA	76075.977	1	631.025972	2.60E-134
Center	18981.98	2	78.724735	1.34E-34
BWA	4239.435	5	7.032952	1.44E-06
Race	150187.094	5	249.150811	6.51E-249
Residuals				
GATK Indel VQSR TS 99.0				
	Sum Sq	Df	F value	Pr(>F)
C20X	52930.43	1	153.90042	4.95E-35
WGA	3744887.28	1	10888.62716	0.00E+00
Center	383585.43	2	557.65614	4.53E-228
BWA	169507.9	5	98.57217	2.76E-101
Race	146904.86	5	85.42806	7.59E-88
Residuals	2876257.21	8363		

ANOVA results table. Sum. Sq., Sum of Squares; Df, Degrees of Freedom

S10 Table. Variance in LOF indel burden explained by technical covariates for each indel filtering approach

S. Table 12

	WGA LOF indel AC/ exon area	DNA LOF indel AC/ exon area
A/T Homopolymer/ exon area	-0.16	-0.11
G/C Homopolymer/ exon area	0.19	0.03

LOF indel allele counts were calculated for each gene separately for WGA and DNA samples. Both allele counts and homopolymer region counts were normalized by gene by dividing by coding exon length in base pairs.

S12 Table. Spearman correlation between LOF indel burden and homopolymer content
